# High-Throughput Genotyping of Resilient Tomato Landraces to Detect Candidate Genes Involved in the Response to High Temperatures

**DOI:** 10.3390/genes11060626

**Published:** 2020-06-07

**Authors:** Fabrizio Olivieri, Roberta Calafiore, Silvana Francesca, Carlo Schettini, Pasquale Chiaiese, Maria Manuela Rigano, Amalia Barone

**Affiliations:** 1 Department of Agricultural Sciences, University of Naples Federico II, Portici, 80055 Napoli, Italy; fabrizio.olivieri@unina.it (F.O.); roberta.calafiore@unina.it (R.C.); silvana.francesca@unina.it (S.F.); chiaiese@unina.it (P.C.); mrigano@unina.it (M.M.R.); 2 ALMA SEGES Soc. Coop., Eboli, 84025 Salerno, Italy; carlo@almaseges.com

**Keywords:** genotyping-by-sequencing, heat tolerance, tomato landraces, wild-species, yield-related traits

## Abstract

The selection of tolerant varieties is a powerful strategy to ensure highly stable yield under elevated temperatures. In this paper, we report the phenotypic and genotypic characterization of 10 tomato landraces to identify the best performing under high temperatures. The phenotyping of five yield-related traits allowed us to select one genotype that exhibits highly stable yield performances in different environmental conditions. Moreover, a Genotyping-by-Sequencing approach allowed us to explore the genetic variability of the tested genotypes. The high and stable yielding landrace E42 was the most polymorphic one, with ~49% and ~47% private SNPs and InDels, respectively. The effect of 26,113 mutations on proteins’ structure was investigated and it was discovered that 37 had a high impact on the structure of 34 proteins of which some are putatively involved in responses to high temperatures. Additionally, 129 polymorphic sequences aligned against tomato wild species genomes revealed the presence in the genotype E42 of several introgressed regions deriving from *S. pimpinellifolium*. The position on the tomato map of genes affected by moderate and high impact mutations was also compared with that of known markers/QTLs (Quantitative Trait Loci) associated with reproductive and yield-related traits. The candidate genes/QTLs regulating heat tolerance in the selected landrace E42 could be further investigated to better understand the genetic mechanisms controlling traits for high and stable yield trait under high temperatures.

## 1. Introduction

Global warming and the increased food demand due to the world population growth rate are two critical threats to the quality of human life. The rising rate in global temperatures predicted for the next few years [1,2] can lead to harvest losses affecting food availability. Heat stress (HS) is one of the abiotic stresses that mainly alters plant life cycles. HS can deeply affect photosynthesis, metabolic activities, cellular divisions, and protein folding. Moreover, in different plant species, HS can affect all the reproductive phases, as documented in cereals, oil crops, pulse crops, and vegetables crops [3]. Among these, the tomato is regarded as one of the most important horticultural crops for its economic value and for its beneficial nutritional properties and it is largely used in the Mediterranean diet. It has been demonstrated that, in tomatoes, when temperatures exceeds 35 °C, all the reproductive stages, from pollen formation and viability to fruit set [4], are adversely affected, causing yield reductions [5]. Therefore, the expected increase of 1–3 °C in average diurnal temperatures over the next few years may greatly reduce the total production in tomatoes [6,7]. Besides the choice of efficient agronomic practices, the selection of heat tolerant genotypes, which exhibit good performances under different environmental conditions, could represent a strategic approach to address this issue. As such, the local landraces and the wild ancestors of domesticated crops constitute the best source of genetic variability useful for identifying genes and/or QTLs (Quantitative Trait Loci) involved in heat stress responses [8]. Genetic variability has been lost during the selection of commercial varieties, since these lines have been generally selected considering only their high yield and their attitude for processing. The consequent reduction of genetic diversity, and the extensive use of F_1_ hybrids selected for commercial traits, decreased the variability in terms of fruit quality and resistance to abiotic stresses [9,10]. Therefore, landraces and wild ancestors of crop varieties can be used to improve the traditional varieties and also to investigate molecular mechanisms controlling tolerance to abiotic stresses, including heat stress. In order to characterize these genotypes under high temperatures, rigorous phenotyping in different environmental conditions combined with whole-genome genotyping must be carried out. Currently, genotyping can be realized using the Next Generation Sequencing technologies that offer a solution for identifying thousands of markers greatly reducing the sequencing costs [11]. These technologies, including Genotyping-by-sequencing (GBS), are powerful tools to identify DNA polymorphisms with results that can be easily applied in plant breeding [12,13]. If the whole genome sequence of reference organisms is already available, an approach like RAD-seq (Restriction Site-Associated DNA sequencing) is highly cost-effective. This technique can sample about 200,000 SNPs (Single Nucleotide Polymorphisms) with the same coverage depth and at nearly 35-fold lower costs compared to the Whole Genome Sequencing of the same number of individuals [14]. Another important feature of GBS technology is that it can be used for identifying domestication-related genes by comparing the genomes of crop species with those of wild ancestors [15].

The aim of the present work was to phenotype 10 tomato landraces grown under elevated temperatures for yield-related traits by comparing different environmental conditions and different years. These landraces were also genotyped in order to exploit their genetic variability. Herein, the mutations (SNPs and InDels) identified were annotated and a prediction of their effect on protein functions and structures was carried out. The phenotypic and genotypic data recorded in the present work were used for selecting stable heat-tolerant tomato landraces and for identifying candidate genes associated with thermotolerance. In the future, these data could be used as a starting point in breeding programs to constitute F_1_ hybrids and to better understand the genetic mechanisms underlying heat tolerance in tomatoes.

## 2. Materials and Methods 

During the summer of 2016 and 2017, 10 tomato landraces (coded E) and two hybrid genotypes, available at the University of Naples, Department of Agricultural Sciences, were grown in two regions of Southern Italy (Campania and Puglia) usually characterized by elevated temperatures during the summer season. Details on the genotypes are shown in Supplementary Table S1 and are hosted at LabArchive repository (http://dx.doi.org/10.6070/H4TT4NXN). The two hybrids DOCET and JAG8810 have been used as control genotypes since they perform good yields under high temperatures (from Giulio Bile, MONSANTO, personal communication). The 10 tomato genotypes coded E were selected from a wide tomato germplasm collection previously characterized for fruit morphology and nutritional traits and evaluated under elevated temperature conditions [16,17,18]. Plants were grown in a completely randomized block with three replicates per genotype and 10 plants per replicate. Seeds were sown in seed trays kept in a greenhouse in the third decade of April and seedlings were transplanted to open fields by the end of May or the beginning of June, with a one-month delay compared to the usual transplanting period of the growing area. This delay imposed a high-temperature condition during flowering and fruit setting. The management practices carried out in each location followed the standard agronomic practices of each cultivation area. The insecticides and fungicides were applied to the plants according to local practices and recommendations. Urea phosphate fertilizer (40 kg ha^−1^) was applied to the soil before transplanting. Tillage treatments included plowing that was followed by one/two millings. Weeding and ridging were also carried out. Recommended levels of N (190 kg ha^−1^) and K (20 kg ha^−1^) were applied through irrigation. During cultivation, plants were irrigated as required. During the whole growing season, climatic data were recorded using the weather station VantagePro2 (Davis Instrument Corporation).

### 2.1. Phenotypic Evaluation

Five phenotypic traits associated to yield performances were evaluated: number of flowers per inflorescence (NFL), fruit set (FS), total number of fruits per plant (TNF), fruit weight (FW), and yield per plant (YP). NFL was evaluated counting the number of flowers per inflorescence from the second to the fifth truss on three plants per replicate, whereas FS was evaluated by counting the number of flowers setting fruit out of all flowers of each inflorescence from the second to the fifth truss on the same three plants per replicate. TNF was calculated by summing all fruits collected from all plants of the three replicates. The final value was divided for the number of total plants. FW was measured by weighting at least 50 fruits collected from all the plants and dividing the value for the number of total fruits used. Lastly, YP was evaluated using the total weight of all collected fruits and dividing it for the number of total plants of the three replicates. These three last parameters were determined at the red ripe fruit stage.

### 2.2. Statistical Analysis

In order to better understand the genotype by location by year interactions for all phenotypic traits, an ANOVA analysis was carried out, where the genotype, location, and year were considered fixed factors. The stability performance of each genotype in terms of yield per plant was evaluated using a linear regression model [19]. In this model, the b_i_^v^ value (coefficient of regression) was calculated for each genotype, considering the average yield of each field on the y-axis and an environment index (*EI*), estimated as Equation (1), on the x-axis.

(1) $$EI=\sum_{i=1}^{v}\frac{Y_{ij}}{v}-(\overset{v}{\underset{i=1}{\sum }}\overset{l}{\underset{j=1}{\sum }}\frac{Y_{ij}}{v_{l}},\;\overset{l}{\underset{j=1}{\sum }}EI=0)$$

where *Y_ij_* is the yield of the *i^th^* genotype (*i* = 1, 2, 3, …, *v*) at the *j^th^* location (*j* = 1, 2, 3, …, *l*). The coefficient of regression (b_i_^v^) describes the linear correlation between the yield of the single genotype and the different environments. In this linear regression model, a b_i_^v^ = 1 was considered the optimum value describing the phenotypic stability of a trait. The standard deviation of b_i_^v^ coefficients was calculated on the whole population to establish threshold borders of confidence for selecting stable-yielding genotypes. The average deviation from linearity (ADL) (Equation (2)) was estimated as:

(2) $$ADL=\frac{\sqrt{\sum_{i=1}^{l}{\left(\mu_{Qij}-\mu_{Lij}\right)}^{2}}}{\overset{=}{\mu }}$$

where *µ_Q_* is the predicted yield mean in a best-fit non-linear model (quadratic model) of the *i^th^* genotype at the *j^th^* location, *µ_L_* is the predicted mean in a linear model of the *i^th^* genotype at the *j^th^* location, and $\bar{\mu }$ is the average yield of *i^th^* genotype in different locations. The ADL describes the average of deviation from linearity. When the ADL parameter calculated for each b_i_^v^ resulted in a significantly different value from zero, the coefficient of regression was considered inconsistent for selecting high-yielding and stable-yielding genotypes. Lastly, the coefficient of determination (*r^2^*) indicated the accuracy of the statistical model. In this way, *r^2^* > 0.50 is a condition for the accuracy of the regression model, essential to identify high—and stable—yielding genotypes. The genotypes showing a YP mean higher than the grand mean, b_i_^v^ ≤ 1, *r^2^* > 0.50 and ADL is not significantly different from 0. These were regarded as high—and stable—yielding genotypes. Multiple statistical test analyses were carried out using the software SPSS version 23 (IBM Corp., Chicago, IL, USA).

### 2.3. Genotyping Analysis

Total genomic DNA was extracted from 100 mg of young leaf tissue from all the genotypes using the DNeasy plant mini kit (Qiagen, Hilden, Germany). The DNA concentration was determined using the Qubit fluorometer (Invitrogen, Carlsbad, CA, USA) while the 260/280 and 260/230 ratios were measured using the Nanodrop spectrophotometer (Thermo Fischer, Waltham, MA, USA). For DNA sequencing, samples (1 µg in 30 µL of sterile milliQ water) were used to prepare libraries for the ddRAD sequencing, as described in Peterson et al. [20] with minor modifications. MboI and SphI enzymes were used for restriction digestion and fragments sequenced with the V4 chemistry paired end 125 bp mode on the HiSeq2500 instrument (Illumina, San Diego, CA, USA). Demultiplexing of raw Illumina reads was carried out using Stacks v2.0 [21]. Alignment to the reference genome of *Solanum lycopersicum* (Tomato Genome version SL3.0, available at the Solgenomics Network, www.solgenomics.net) was performed using BWA-MEM (Burrow-Wheeler Alignment - Maximal Exact Matches) [22] with default parameters and selection of uniquely aligned reads (i.e., reads with a mapping quality higher than 4). The detection of all the covered loci from the aligned reads and the variant calling were performed using the reference-independent Stacks pipeline. At the end of these processes, two VCF (Variant Call Format) files (one for the SNPs dataset and one for the InDels) were obtained and subjected to a filtering procedure using VcfTools v.0.1.13 (http://vcftools.sourceforge.net). Filtering parameters were set as follows: maximum missing value = 0.50 and minimum mean of Depth of Coverage (min-mean DP) = 5. All the mutations that were not polymorphic among the 12 analyzed genotypes were manually eliminated since they showed the same alternative allele with respect to the reference genome of the cv. Heinz. The visual display of sequencing data was performed using CircosVCF webtool [23] by generating a circus plot from VCF data (http://212.150.245.226/~tools/CircosVCF/). Then, in order to estimate the genetic distance among the genotypes, pairwise comparisons were performed by calculating the Identity-by-State (IBS) allele-sharing values using the software PLINK v.1.90b5.2 [24] and represented by the IBS distance matrix, where values tending to 0 indicated higher genetic distances. Gene annotation and prediction of the possible effects of the SNPs and InDels mutations on proteins’ structure were evaluated by software SNPeff v. 4.3T (http://snpeff.sourceforge.net/), using the SL3.0 version of the tomato genome to define the predicted SNPs’ effect and the annotation version iTAG3.2 to identify the affected protein. Four tags were used to estimate the putative impact of SNPs and InDels on genes’ expression and proteins’ translation. This is high for the variants assumed to have a disruptive impact on the protein function, moderate for non-disruptive variants that could change the protein effectiveness, low for the synonymous variants that should not affect the protein behavior, and, lastly, modifier effect for variants located in non-coding regions. Lastly, in order to investigate the origin of the genetic variability detected in the genotype E42, starting from the dataset of filtered InDels, an alignment was performed against genomic sequences of wild species publicly available (*S. chilense*, *S. galapagense*, *S. pennellii*, *S. peruvianum*, and *S. pimpinellifolium*). A global alignment was performed using the wild tomato species databases (available at ftp://ftp.solgenomics.net/genomes/) and the software Blast Genome Workbench v. 3.1.0 (https://www.ncbi.nlm.nih.gov/tools/gbench/). The screening of InDel loci was carried out by selecting only the polymorphisms showing an insertion or deletion of at least five nucleotides. The SSR-InDels were selected based on differences in the number of repeats (at least one repeat). Lastly, a sequence of about 100 bp, including the InDel mutation, was used as a query for the analysis. We considered putative wild species-derived loci that showed 100% identities on the InDel region and more than 90% identities in the whole 100-base pair sequence.

## 3. Results

The 10 tomato genotypes analyzed in this work were previously characterized for fruit quality and nutritional traits and were selected for their good performances under high temperature conditions [16,17,18]. In this case, we report their phenotypic characterization carried out to better define their performances under elevated temperatures. Five yield-related phenotypic traits were evaluated in the years 2016 and 2017 in two different regions of Southern Italy (Campania and Puglia), usually characterized by high temperatures during the tomato growing season. In order to verify the climate conditions, day/night maximum temperatures were monitored during the four analyzed seasons (Figure S1). In the Campania fields (coded C2016 and C2017, for the two years), the maximum diurnal temperature never reached 35 °C, whereas, in the Puglia fields (coded P2016 and P2017, for the two years), it was over 32 °C for approximately half of the growing season and peaked over 35 °C for 15 days in the year 2017, reaching 38–39 °C. On average, the maximum nocturnal temperatures recorded in the Campania fields were higher in 2016 (24.9 °C) than in 2017 (23.5 °C). Moreover, in the Puglia fields, the maximum nocturnal temperatures recorded were higher than in Campania.

### 3.1. Phenotypic Analysis

**Figure 1:**
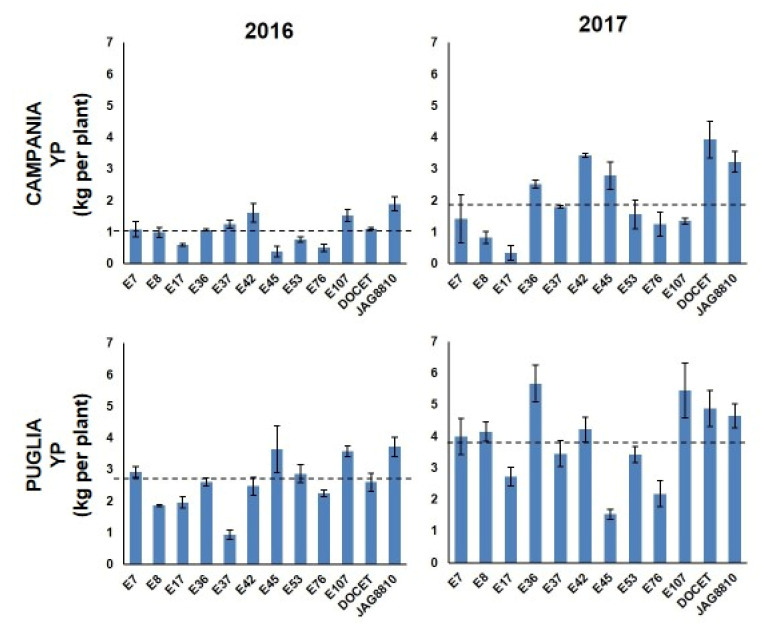
Yield per plant (YP) observed in the 12 tomato genotypes in Campania and in Puglia in the years 2016 and 2017. Dashed lines indicate the mean values.

The landraces’ performances evaluated in all the fields were compared to those of two tomato hybrids (DOCET and JAG8810) that are highly productive under high temperature conditions. A high variability of phenotypic data was observed for almost all the traits in the different growing locations and in the different years (Table S2). For some traits, most genotypes showed comparable levels in the four trials, with some exceptions (Figure S2 and Figure 1). For example, in most genotypes, except E17 and E53, the highest number of flowers per inflorescence (NFL) was observed in Campania in 2016. As for the fruit set (FS), in both fields, most genotypes showed values higher in 2016 than in 2017. Considering the Campania fields, only E42 and E45 showed values higher in 2017 than in 2016. In Puglia E17, E53, E107, and DOCET showed a better FS performance in 2017 when compared with 2016. Genotypes with FS values higher than 75% were observed in 2016 in Campania (~42% of genotypes) and in Puglia (~25% of genotypes). Regarding the number of fruits per plant (TNF), the highest values were observed in Puglia in 2017 for all the genotypes, whereas, in the other three fields, the values of TNF were more or less similar. In all the experimental fields, E17 and E42 showed the lowest and the highest value of TNF, respectively. The fruit weight (FW) showed a similar trend in all the fields with genotypes exhibiting a general reduction of FW in 2017 compared to 2016, and, in all cases, the lowest and the highest FW were recorded in E42 and E17, respectively. Lastly, yield per plant (YP, Figure 1) showed a wide range of values in the different locations with the best performances mostly registered in Puglia in 2017 and the worst ones registered in Campania in 2016. In particular, in Campania in 2016, E45 and the hybrid JAG8810 showed the lowest (0.38 kg/plant (pt)) and the highest (1.89 kg/pt YP, respectively, and the population mean was 1.06 kg/pt (Table S2). In the second year in Campania, the YP values ranged from 0.34 kg/pt (E17) to 3.93 kg/pt (DOCET) with a mean value of 2.04 kg/pt. In Puglia in 2016, YP values ranged from a minimum of 0.94 kg/pt (E37) to a maximum of 3.72 kg/pt (JAG8810) with a mean value of 2.62 kg/pt. In the second year, the values in Puglia ranged from 1.53 kg/pt (E45) to 5.67 kg/pt (E36) with a mean value of 3.86 kg/pt. Student’s *t*-test was performed to estimate statistical differences between the landraces and the heat tolerant control hybrids (DOCET and JAG8810) for all the evaluated traits in each field. As reported in Table 1, a high variability was observed in terms of significant differences for all traits. For NFL, the heat tolerant controls showed a significantly lower number of flowers per truss compared to all the landraces, except the two genotypes E45 and E107. As for FS, six genotypes exhibited values significantly higher than the controls in different fields, whereas E107 was always comparable to both the controls and E17 showed mostly lower values. The genotype E42 always exhibited TNF values higher than the controls. Lower levels were instead always recorded in E17. For all the genotypes, FW was lower than the controls, except than for E17 and E53, and this well reflects the different fruit size of the analyzed genotypes. Lastly, all genotypes showed YP values lower than the controls, except E7 and E42 that, despite their lower fruit size compared to DOCET and JAG8810, showed values of YP comparable to that of the control genotypes. Since differences in weather conditions, such as atmospheric humidity, temperatures, and wind, can affect the phenotypic responses of plants during the growing season, a three-way ANOVA analysis was carried out to evaluate the genotype by location by year interaction (G × L × Y) (Table S3), considering all the 12 genotypes. For each trait, the contribution of variance to each source of variation, expressed as the Total Sum of Square percentage (TSS%) was also evaluated. In four out of five phenotypic traits, the factor genotype showed the highest values of TSS%, ranging from 31.16% for NFL to 84.50% for FW. Only for YP, the effect of the location (TSS% = 35%) was higher than that of the genotype (TSS% = 19.72%), and only TNF did not show significant G × L × Y interaction. The Pearson’s correlation was also calculated among the phenotypic traits recorded in each location (Table S4). As a whole, an expected negative correlation was always detected between FW and TNF with the *r*-value ranging from −0.63 to −0.45. TNF was positively correlated with YP in Campania in both years (*r* = 0.56 in 2016 and *r* = 0.53 in 2017, respectively) and in Puglia in 2017 (*r* = 0.51). When considering the significant correlation indexes between YP and the four traits NFL, FS, TNF, and FW detected for each genotype (Table S5), highly positive correlation coefficients were detected between TNF and YP (in most cases, *p* < 0.001). 

### 3.2. Selection of Stable-Yielding Genotypes

**Figure 2:**
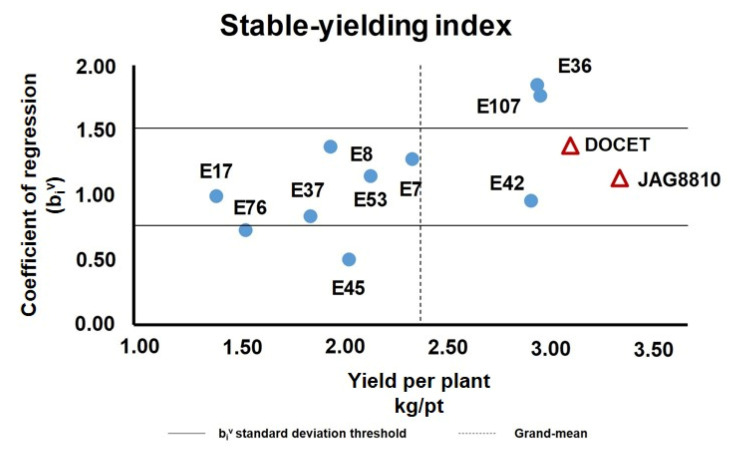
Diagram showing the stable-yielding index evaluated in the 12 genotypes analyzed. Yield/pt (YP) averages of each genotype over four environments were plotted against their coefficient of regression, calculated as reported in Supplementary Table S6. The horizontal lines delimitate the range of variation of the b_i_^v^ index between 0.77 and 1.57. The dashed vertical line indicates the grand mean YP value.

A linear regression method was used to select high-yielding and stable-yielding genotypes by determining the coefficient of regression (b_i_^v^) value (Figure S3 and Table S6). This value ranged from 0.51 (in E45) to 1.78 (in E107) and reached the optimum b_i_^v^ ≃ 1 in the genotypes E17 and E42 (Table S6). Three out of 12 genotypes (E8, E37, and E45) showed an average deviation from a linearity (ADL) value significantly higher than zero, underlying an inconsistent value of b_i_^v^. As reported in Figure 2, about 67% of genotypes showed a b_i_^v^ ranging between the standard deviation values of 0.77 and 1.57 around the mean of 1.17. 

The YP grand mean of the group in different locations was calculated (2.39 kg/pt) to establish which genotypes showed a YP pooled mean (calculated considering the values of four locations) higher than the group grand mean (calculated considering the values of all genotypes in all locations). In this way, ~42% of genotypes (five out of 12) showed a YP pooled mean higher than the grand mean. Among these genotypes, the landrace E42 was identified as a stable yielding genotype (b_i_^v^ = 0.96, YP = 2.93 kg/pt, ADL = 0, *r^2^* = 0.72). In fact, notwithstanding the other two genotypes (E36 and E107), a YP pooled mean was higher than the grand mean. In addition, their coefficient of regression b_i_^v^ was higher than 1, which suggested that these genotypes achieved high yield only in favorable environments. 

### 3.3. GBS Analysis

**Figure 3:**
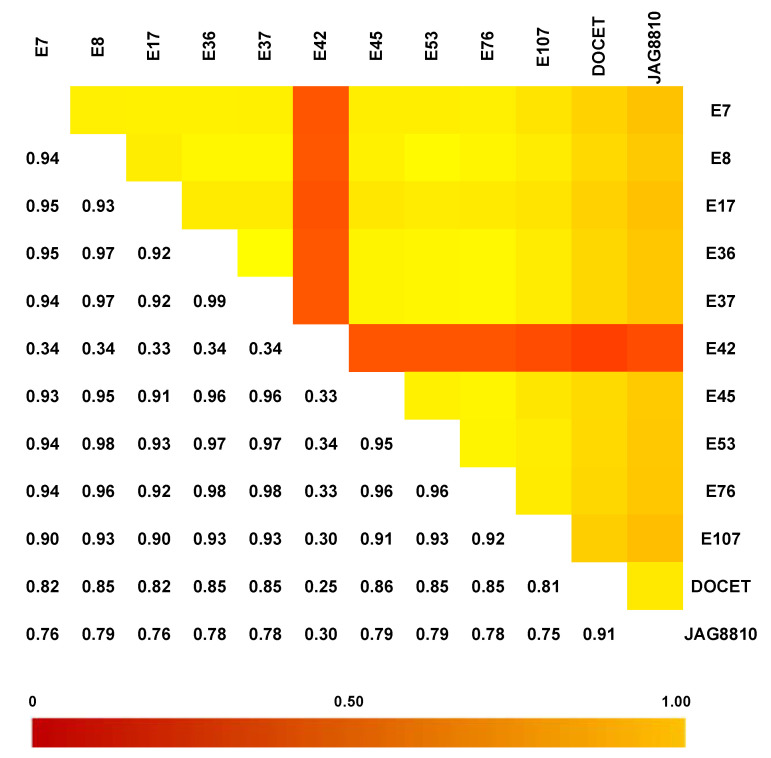
Identity-by-state (IBS) distance heat matrix among the 12 genotypes considering the dataset of 22,594 SNPs. In each pairwise comparison, values near 1 (yellow color) indicate low genetic distance whereas values near 0 (red color) represent high genetic distance.

**Figure 4:**
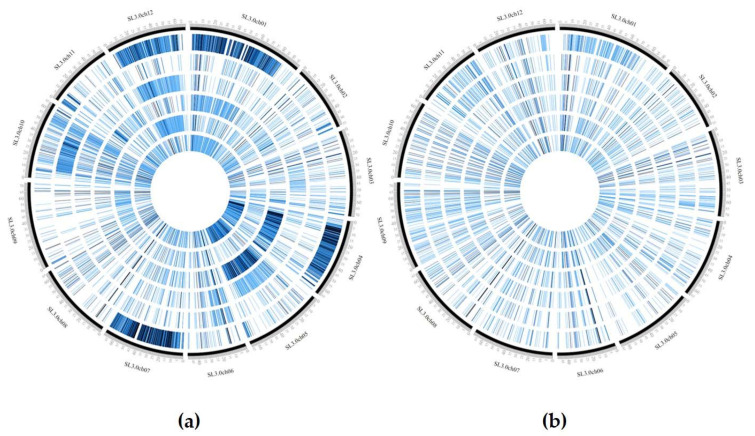
Distribution of SNPs density in the evaluated genotypes using CircosVCF software. The 12 genotypes were divided in two groups based on SNPs density. The genotypes from the external to internal ring are: (**a**) E42, E107, E17, JAG8810, E7, and DOCET, (**b**) E45, E76, E37, E36, E53, and E8.

The GBS analysis performed on the 10 landraces and the two hybrids using ddRAD Illumina technology produced 15,577,778 reads, and an average of 1,112,698 reads per sample. The SNP calling procedure revealed 103,859 unfiltered SNPs/InDels. Following the filtering settings, the number was reduced to 22,594 SNPs and 3519 InDels. In addition, SNP calling revealed that most SNPs detected were homozygous (93.1%) in both the reference (83.0%) and alternative allele (10.1%). Low values were recorded for SNPs/InDels in a heterozygous condition (6.1%) and only 0.5% of missing data were observed. As expected, the two hybrids DOCET and JAG8810 showed the highest percentage of heterozygous SNPs/InDels (21.6% and 31.7%, respectively). By a pairwise comparison of all the genotypes (Figure 3), based on IBS (Identity-by-state) values, it was possible to observe that the genotype E42 was the most distantly related from all the other genotypes with an IBS value of approximately 33–34%, whereas, in all the other comparisons involving the landraces, the index was higher than 90%. When IBS was calculated in comparisons involving the two control hybrids DOCET and JAG8810, the values were around 80% and 70%, respectively. In addition, considering the filtered 22,594 SNPs, which map throughout the tomato genome, we observed that the genotype E42 showed the highest SNPs density on chromosomes 1, 4, 7, and 12 whereas both the hybrids exhibited higher SNPs’ density on chromosomes 4 and 5 (Figure 4).

In order to exploit the genetic variability exhibited by the different landraces, the number of private SNPs and InDels was also calculated for each genotype (Table 2). In most cases, private SNPs were distributed throughout the whole genome and the minimum number of private SNPs (11) was recorded in genotypes E36 and E37, whereas the minimum number of private InDels (14) was recorded in E36. The stable-yielding landrace E42 showed 11,050 private SNPs with the highest density on chromosomes 1 (3865), 4 (745), 7 (4317), and 12 (1773), corresponding to about 97% of total private SNPs for this genotype. Moreover, E42 showed 1640 private InDels, corresponding to about 47% of the whole InDels dataset.

In order to understand the effects of the mutations on gene expression and protein translation, the annotations of the mutated genes and the prediction of the mutation effect were investigated (Table 3). Out of 22,594 SNPs, the variants with a modifier effect on the protein were 97.7%. Those with a low and moderate effect were approximately 1.0%. In the same way, out of 3519 InDels, 97.4% were InDels with a modifier effect, whereas the mutations with low and moderate impact were 0.7% and 1.3%, respectively. All the detected SNPs and InDels targeted approximately 6000 genes. In addition, 15 SNPs and 22 InDels showed a disruptive or high impact effect on the corresponding proteins.

The 34 genes affected by these variations (Table 4) are distributed on the 12 chromosomes and some of them could be involved in abiotic stress responses such as the genes *Solyc02g087680* and *Solyc02g087690* coding for a FACT complex subunit SSRP1 and *Solyc12g044645* coding for the transcription factor AP2/B3 family protein. Interestingly, 14 different variants (7 SNPs and 7 InDels) resulted private for the genotype E42. Moreover, concerning the variants with predicted moderate effects on protein structure (Table S7), 283 different variants were identified (239 SNPs and 44 InDels) to affect 201 genes, and 43.8% were private variants for the genotype E42. 

Lastly, in order to investigate the origin of the high genetic variability of the genotype E42, starting from the dataset of 3,519 filtered InDels, an alignment of the selected InDels was performed against genomic sequences of wild species publicly available (*S. chilense*, *S. galapagense*, *S. pennellii*, *S. peruvianum* and *S. pimpinellifolium*). For this purpose, 129 InDels were chosen and, for each of them, a 100-base pair sequence including the InDel was selected for the BLAST analysis. Among these, 27 InDels consisting of SSR (Simple Sequence Repeats) markers (coded S in Table S8) and 36 InDels (coded N in Table S8) showed high percentages (>90%) of identity with the 100-base pair analysed sequence from *S. lycopersicum* and 100% of identity with the InDel target region of wild species’ genomes. Furthermore, 25 InDels-SSR (93%) and 22 InDels (61%) were identified as putatively derived from *S. pimpinellifolium*. The other two InDels-SSR and 14 InDels loci showed high identity percentages when aligned against the genomes of *S. chilense* and *S. galapagense*.

## 4. Discussion

Facing damage caused by abiotic stresses, such as drought and heat stress, represents a great challenge for agricultural science in the new century. Exploiting genetic resources is one tool to achieve this objective, in combination with optimized growing and management practices. In the present work, 10 tomato landraces have been tested for two years in two different open fields under high temperature conditions. As expected, the climatic parameters were highly variable in the four experimental conditions tested, including not only diurnal and nocturnal temperatures, but also relative humidity, wind, and rain. Other environmental factors, such as soil composition and texture, might have influenced the performances of the tested genotypes in the four trials. However, the phenotypic analyses performed on the 10 landraces allowed us to identify those that exhibited the best stable performances in terms of yield under a wide range of conditions, including high temperatures. Usually, heat tolerance has been evaluated under fixed climatic conditions in greenhouses or growth chambers [25,26,27]. However, these conditions do not reflect those observed in the fields during the tomato growing seasons. In most studies, the impact of high temperatures on plants has been evaluated using fixed diurnal and nocturnal temperatures and applying an extreme short-term stress or a mild long-term stress [28,29,30,31,32]. In the present study, we considered yield as the most reliable trait to assess heat tolerance, even though other yield components, such as NFL, FS, TNF, and FW, were evaluated. These yield-related traits were reported to impact adaptation of crops to unfavourable abiotic conditions [33]. Moreover, it is important to highlight that, in some cases, a key role on final yields under abiotic stresses has also been attributed to the strength of the source-sink communication, as reported in different plant species [34]. This important aspect will be further investigated in future works. Contrary to studies carried out under controlled conditions, we did not observe a drop in FS values despite the high temperatures recorded in the fields. This likely could be explained by the fact that the field temperatures are not fixed but fluctuate during the whole day, often reaching peaks over the optimal range. As for YP, among the tested genotypes, the landrace E42 was the most stable one, as shown by the values of stability parameters [19,35]. E42 was the only landrace that exhibited yield performances comparable to those of the two F_1_ hybrids used as control (DOCET and JAG8810), even showing a higher stability compared to them. The level of YP observed in all the genotypes, across the four experimental fields, showed that this trait was highly correlated with the TNF value, and the latter was mainly correlated with the NFL value. This occurred independently of the different FW values recorded herein, which ranged from the low values recorded in E42, characterized by very small fruits, to the high values recorded in E17, characterized by big-sized fruits. These results are in contrast with data reported by Ayenan et al. [36], who found that tomato yield improvement under high temperatures depends on both fruits’ weight and number. In our study, only TNF correlated positively to the final yield of all the tested genotypes. In the genotype E42, a high NFL value was also registered and this trait likely affected the high TNF values and, consequently, the YP values observed. Therefore, the genotype-dependent trait NFL likely was the most important one to determine the good and stable performances of E42 under high temperature conditions.

The genotyping analyses performed in this scenario demonstrated that E42 has a high genetic variability compared to the other genotypes investigated. This could be due to the breeding history of this genotype, which likely included crossing events with tomato wild species. A group of selected InDel mutations aligned against the genome of five wild species revealed that in the genotype E42 putative introgressed regions, mainly deriving from the species *S. pimpinellifolium*, were mapped on chromosomes 1 and 7. The presence of these and/or other wild regions has potentially contributed to confer the observed thermotolerance to E42. It has been reported that the wild tomato species usually show high pollen viability and pollen number even under heat stress conditions [37]. Moreover, it has been demonstrated that some accessions of *S. pimpinellifolium* exhibit a good level of thermotolerance [38,39].

Following the high-throughput genotyping of the landraces analysed, we demonstrated that 37 and 283 mutations were mapped within coding regions and exhibited a high and moderate impact on the corresponding proteins, respectively (Figure S4). Therefore, they were considered the most critical ones for determining the tolerance to high temperatures. It is noteworthy that 16% of the mutations classified as modifier was mapped into gene regions exhibiting regulatory functions. The role of such mutations could be further investigated by a transcriptional analysis at specific developmental stages and under controlled and stressed conditions. 

**Figure 5:**
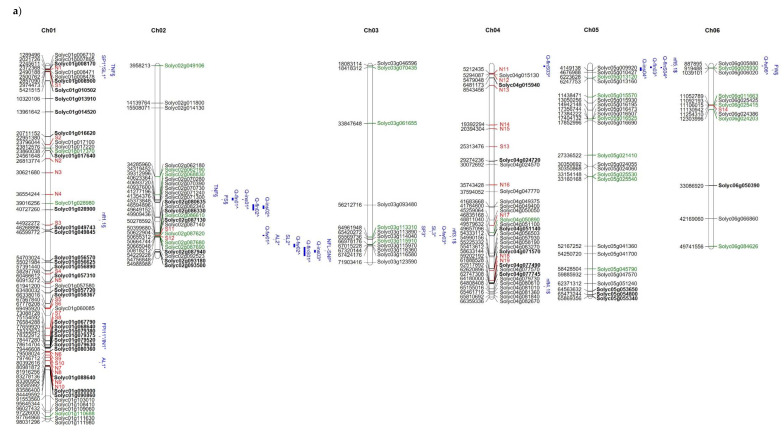

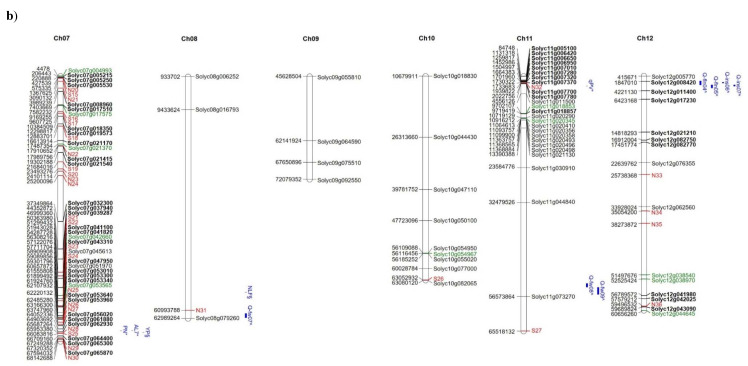
Distribution on the 12 tomato chromosomes of genes affected by mutations with high (green colour) or moderate (black colour) impact, (**a**) chromosomes from 1 to 6, and (**b**) chromosomes from 7 to 12. The private mutations of the genotype E42 are shown in bold, whereas, in red, are reported as the markers with alleles deriving from wild species. The position of markers/QTLs reported in the literature are shown in blue (§ from Ruggieri et al., 2019 [18], * from Xu et al., 2017 [31], $ from Hernandez-Bautista et al., 2016 [40], and ^ from Zhang et al., 2018 [41]).

The distribution of high and moderate mutations on the 12 tomato chromosomes is reported in Figure 5 together with the position of markers/QTLs reported in literature [18,40,41] as associated with the traits analysed in the present study such as NFL, TNF, FW, and YP. In addition, the position of markers/QTLs associated with reproductive traits potentially involved in the response to high temperatures [31] are localized on the map. It is evident that, in some chromosomal regions (see chromosomes 1, 2, 7, 12), genes showing private mutations in E42 co-localize with QTLs/markers associated with reproductive traits, such as pollen viability, pollen number, style protrusion and length, anther length, inflorescence number, number of flowers, or total fruit. It is also evident that, in the same regions, are localized markers that carry alleles deriving from wild species. For example, a QTL associated with yield and mapping at the end of chromosome 7 co-localizes with many private markers in E42 and markers deriving from *S. pimpinellifolium*. Since the good yield performances of E42 under high temperatures were mostly attributed to the high NFL and to the high TNF observed, we hypothesized that one or more genes influencing the number of flowers could exhibit informative mutations in E42. It has been reported that two QTLs carrying a *S. pimpinellifolium* allele and mapping on chromosomes 2 and 4 affect the number of flowers in tomatoes [41]. Moreover, very recently, a QTL displaying a consistent effect on NFL and TNF was located on the distal region of chromosome 2 in a set of RILs and ILs deriving from a cross between *S. lycopersicum* and *S. pimpinellifolium* [38]. In the present study, we found SNPs and InDels in these same regions, which are currently being investigated.

Lastly, searching for genes potentially involved in the response to high temperatures, we could find mutations in four genes with a disrupting impact on the corresponding proteins with three of them showing SNPs/InDels private for the genotype E42. Two genes mapped close to each other on chromosome 2 (*Solyc02g087680* and *Solyc02g087690*) and both coded for members of the FACT subunit complex SSRP1 protein family. It has been reported that, in Arabidopsis, this protein is an epigenetic regulator of seed dormancy and it is also involved in anthocyanin biosynthesis, and in high-light and UV stresses responses [42]. The third private mutation for E42 mapped in the gene *Solyc07g042660*, which codes for an RNA helicase, known to be involved in the responses to different abiotic stresses. Moreover, the expression levels of several DEAD-box helicases are regulated in response to environmental stresses, including salt, light, or temperature stresses [43,44]. Lastly, two mutations were detected in the gene *Solyc12g044645* coding for an AP2/B3 transcription factor family protein. This gene is homologous to *At1g51120.1* identified in *A. thaliana*, which codes for a member of the RAV superfamily involved in seed germination, plant development [45], and responses to abiotic stresses, including salinity and drought [46]. Among genes carrying mutations with a moderate effect, five putatively involved in the heat stress response were also identified. The first gene, *Solyc01g017220,* coding for an ATP synthase epsilon chain, is reported to be involved in drought stress responses [47]. The genes *Solyc02g070280*, which codes for a protein involved in the transport of amino acids during the pedicel abscission [48], and *Solyc07g053640*, which codes for an arabinogalactan-protein that could be involved in abscission events [49], might impact on FS. This is a yield-related trait highly affected by high temperatures. On chromosome 8, a missense variant was identified in *Solyc08g079260*, coding for a Hsp70 with a tetratricopeptide domain. Lastly, *Solyc12g043090* codes for a trihelix transcription factor, which is involved in different stress responses. It also showed variations with moderate effects [50]. The mutations identified in *Solyc07g053640* and in *Solyc12g043090* were private for E42.

## 5. Conclusions

We evaluated 10 landraces under high temperatures in multi-environmental conditions to select the best genotypes in terms of stable yield. At the end of this work, we selected the genotype E42 as the best performer due to the high NFL, and, consequently, the high TNF values showed. Following a high-throughput genotyping, E42 also resulted in the most polymorphic genotype, showing some introgressed regions from the wild species *S. pimpinellifolium*. Considering that heat tolerance is a polygenic trait that involves many genes, the identification of candidate genes, obtained by combining the in-depth phenotypic and genotyping analyses carried out in this work, might help dissect this complex trait. In particular, the private SNPs and InDels detected in two QTLs mapping on chromosome 2 could be the starting point to design molecular markers targeting NFL, which is a trait that may contribute to induce a good response to heat. Additional candidate genes identified in this study will be further investigated to detect those involved in a stable response to high temperatures. Once functionally validated, these candidate genes could be useful to design molecular markers and/or apply genome editing techniques with the aim to obtain new tomato genotypes improved for heat tolerance.

## Figures and Tables

**Table 1:** Evaluation of significative differences of five phenotypic traits between the landraces (coded E) and the two control hybrids (DOCET and JAG8810) by Student’s *t*-test (*: *p* < 0.05, **: *p* < 0.01, ***: *p* < 0.001). Red and black asterisks indicate a reduction or an increase of the traits value compared to that of the control genotypes (D = DOCET, J = JAG8810), respectively. (NFL = No. of flowers/inflorescence, FS = Fruit set, TNF = No. of fruit/plant, FW = Fruit weight, YP = Yield/plant. C2016 = Campania field in the year 2016, C2017 = Campania field in the year 2017, P2016 = Puglia field in the year 2016, P2017 = Puglia field in the year 2017).

Trait	Field	Controls	E7	E8	E17	E36	E37	E42	E45	E53	E76	E107	DOCET
NFL	C2016	D				*	**	**		*			
		J					*	**	*			*	
	C2017	D				*	*	*				**	
		J					*	*				**	
	P2016	D			*								
		J	*	**	***	*	*	***			*		
	P2017	D	*			*		**				*	
		J						*	*			**	*
FS	C2016	D							*	*			
		J	*				**			*			**
	C2017	D			*								
		J	**	*	**	**					**		
	P2016	D	**	*	*	**	*		**		**		
		J			***					*			*
	P2017	D			*			*					
		J											
TNF	C2016	D		*	**	*		**	**	*	*		
		J			**			*	**	**	*		
	C2017	D		*	**			***		*		*	
		J			***	**	**	***		*			
	P2016	D	**	**	*	**		***			*		
		J	**	*	*	**		***					
	P2017	D			**			**		*			
		J			**	*		**					
FW	C2016	D	*	**	***	*	*	***		*	**		
		J	***	***	**	**	***	***		**	***		
	C2017	D	***	***	***	***	***	***			***		
		J	*	*	*	*	*	**			*		
	P2016	D	***	***	***	***	***	***		*	***		
		J	**	**	**	**	**	**			*		
	P2017	D			***			**					
		J		*	***			**			*		
YP	C2016	D			***				***	***	**		
		J		*	**	*			**	**	**		*
	C2017	D		**	**		*			*	*	*	
		J		**	***	**	***			*	**	***	
	P2016	D					**					*	
		J		**	**	*	**	*			*		
	P2017	D			*				**		*		
		J			*				**		*		

**Table 2:** Number of private SNPs and InDels reported for each genotype analyzed on the whole dataset of 26,113 filtered markers.

Marker	E7	E8	E17	E36	E37	E42	E45	E53	E76	E107	DOCET	JAG8810
SNP	93	29	288	11	11	11,050	99	47	76	921	51	186
InDel	29	16	55	14	23	1640	41	36	20	83	57	99

**Table 3:** Classification of the effect of SNPs/InDels detected in the analysed genotypes. The impacts of the mutations were categorized with four different tags: high, moderate, and low modifier effect. The total number of genes affected by one or more mutations is also indicated.

Type of Mutation	Total Mutations (no.)	Effect on Protein				Affected Genes (no.)
		High (no.)	Moderate (no.)	Low (no.)	Modifier (no.)	
**SNP**	22,594	15	239	252	22,088	4124
**InDel**	3519	22	44	24	3429	1863
**Total**	26,113	37	283	276	25,517	5987

**Table 4:** List of genes affected by SNPs/InDels mutations with high impact. For each gene, the position in the tomato genome (version SL3.0), the genotypes carrying the mutation, the predicted effect of the mutation, and the protein function are reported.

Gene	Mutation	Position (SL3.0)	Mutated Genotypes	Predicted Effect	Protein Function
*Solyc01g028980*	InDel	39,016,299	E42	stop_gained	Gamma-tubulin complex component
	SNP	39,016,322	E42	stop_gained	
*Solyc02g049106*	SNP	3,958,278	E8, E17, E36, E42, E53, E76, E107, DOCET	stop_gained	Leucine-rich repeat protein kinase family protein
*Solyc02g086610*	SNP	49,909,435	E42	splice_acceptor_variant and intron_variant	Isocitrate dehydrogenase [NADP]
*Solyc02g087620*	InDel	50,622,904	E42	splice_donor_variant, intron_variant	Inositol hexakisphosphate and diphosphoinositol-pentakisphosphate kinase
*Solyc02g087680*	InDel	50,664,745	E42	frameshift_variant	FACT complex subunit SSRP1
*Solyc02g087690*	SNP	50,665,049	E42	stop_lost, splice_region_variant	FACT complex subunit SSRP1
*Solyc03g070435*	InDel	18,418,311	E8, E53, E76, E107	frameshift_variant	Alpha-mannosidase
*Solyc03g061655*	InDel	33,847,933	E7, E8, E17, E36, E37, E42, E53	frameshift_variant	Ribosomal protein S12
*Solyc03g113310*	InDel	64,961,947	DOCET, JAG88100	frameshift_variant	Pseudouridine synthase family protein
*Solyc03g115910*	InDel	66,978,174	E8, E53	frameshift_variant	MADS-box transcription factor
*Solyc04g050890*	SNP	48,811,041	E42	stop_gained	DNA-directed RNA polymerase subunit alpha
*Solyc05g013120*	SNP	6,223,628	E36, E37	stop_gained	Ninja-family protein AFP1
*Solyc05g015570*	SNP	11,438,471	DOCET, JAG88100	start_lost	UDP-glucose 6-dehydrogenase 1
*Solyc05g016525*	InDel	17,404,131	DOCET, JAG88100	frameshift_variant	Core-2/I-branching beta-1
*Solyc05g021410*	InDel	27,336,522	E8, E36, E42, E45, E76, E107, DOCET	frameshift_variant	Histone-lysine N-methyltransferase SUVR5
*Solyc05g025530*	InDel	33,154,148	DOCET, JAG88100	frameshift_variant	DNA-directed RNA polymerase subunit beta
*Solyc05g025540*	SNP	33,160,167	DOCET, JAG88100	stop_gained	Molybdenum cofactor sulfurase
*Solyc05g045790*	SNP	58,428,505	DOCET, JAG88100	splice_donor_variant, intron_variant	Cytochrome c oxidase subunit 2
*Solyc06g005930*	InDel	919,488	E7, E8, E42, E45, E53, E76, DOCET	frameshift_variant	Protein sensitivity to red light reduced 1
*Solyc06g011663*	SNP	11,052,789	E7, E8, E36, E37, E42, E45, E53, E76	stop_gained	Beta glucosidase 25
*Solyc06g025415*	InDel	11,106,015	E7, E8, E17, E36, E37, E42, E45, E107, DOCET	frameshift_variant	Mediator of RNA polymerase II transcription subunit 14
*Solyc06g024203*	InDel	12,303,996	E8, E17, E36, E42, E53, E76, E107, JAG8810	frameshift_variant, splice_region_variant	Peroxidase superfamily protein
*Solyc06g084626*	InDel	49,741,555	E8, E45, E107, JAG8810	frameshift_variant	1-deoxy-D-xylulose 5-phosphate reductoisomerase
	InDel	49,741,558	E17, E45, E107, DOCET, JAG8810	frameshift_variant	
*Solyc07g004993*	InDel	4478	E17, E42, E76, DOCET	start_lost	Phosphatidylinositol N-acetyglucosaminlytransferase subunit P-like protein
*Solyc07g017575*	InDel	7,582,232	E42	stop_gained	Flavin-containing monooxygenase
*Solyc07g021370*	SNP	17,487,354	E42	stop_lost; splice_region_variant	DNA-directed DNA polymerase
*Solyc07g042660*	SNP	56,308,217	E42	splice_acceptor_variant&intron_variant	Helicase SNF2 domain-containing protein
*Solyc07g053565*	InDel	62,107,933	E42	frameshift_variant	NAD(P)H-quinone oxidoreductase subunit 2
*Solyc10g054967*	SNP	56,116,455	E17, E42	stop_gained	Mediator of RNA polymerase II transcription subunit 20-like protein
*Solyc11g018853*	InDel	9,702,107	E42, JAG8810	frameshift_variant	Adenylate isopentenyltransferase
*Solyc11g020345*	SNP	10,916,212	JAG8810	splice_acceptor_variant and intron_variant	Small nuclear ribonucleoprotein family protein
*Solyc12g038540*	InDel	51,497,675	E42	frameshift_variant, splice_acceptor_variant, splice_region_variant, intron_variant	Transducin/WD40 repeat-like superfamily protein
*Solyc12g038970*	InDel	52,525,425	E8, E36, E37, E45, E53, E76, E107, DOCET, JAG8810	frameshift_variant	EMB1873 protein
*Solyc12g044645*	InDel	60,656,260	E42	frameshift_variant	AP2/B3 transcription factor family protein
*Solyc12g044645*	SNP	60,656,273	E42	stop_lost, splice_region_variant	

## Supplementary Materials

The following are available online at https://www.mdpi.com/2073-4425/11/6/626/s1, Figure S1: Temperature variation observed in the four experimental trials during the growing season. Day (red) and night (blue) maximum temperatures are reported for each field. Dashed lines represent critical threshold of 32 °C (red)/26 °C (blue) day/night. (C2016 = Campania field in the year 2016, C2017 = Campania field in the year 2017, P2016 = Puglia field in the year 2016, P2017 = Puglia field in the year 2017). Figure S2: Variation of four phenotypic traits (NFL = No. flowers/inflorescence, FS = Fruit set, TNF = Total number of fruit/plant, FW = Fruit weight) observed in the four experimental trials (C2016 = Campania field in the year 2016, C2017 = Campania field in the year 2017, P2016 = Puglia field in the year 2016, P2017 = Puglia field in the year 2017). Dashed lines indicate the mean values. Figure S3: Linear regression analysis between the yield per plant (YP) and the environmental index (EI) over four experimental trials in 12 genotypes. YP is the mean value of yield over the four trials and the formula for calculating the EI is reported in the Section 2. Figure S4: Heat map showing the distribution on the 12 tomato chromosomes of genes affected by SNP/InDel mutations with high and moderate impact. For each mutation, the reference and alternative alleles are reported, together with the position on the tomato genome (version SL3.0). Table S1: List of the genotypes used for the phenotypic and genotypic screening. Genotypes coded E are 10 tomato landraces collected at the University of Naples Federico II, and previously characterized. The genotypes DOCET and JAG8810 are hybrids kindly provided by Monsanto Italia. For each genotype, data are reported concerning their source, common name, country of origin, product destination, fruit size, and shape. Table S2: Descriptive statistics of five traits evaluated on 10 landraces (code E) and two control hybrids DOCET and JAG8810. Data recorded in: (A) Campania year 2016, (B) Campania year 2017, (C) Puglia year 2016, (D) Puglia year 2017. NFL=No. flowers/inflorescence, FS = Fruit set, TNF = Total number of fruit/plant, FW = Fruit weight, YP = Yield/plant. Table S3: Three-way ANOVA for traits measured on 12 genotypes in two fields and two years. The level of significance (*p*) and the total sum of square percentage (TSS %) are reported. (* *p* < 0.05, ** *p* < 0.01, *** *p* < 0.001, ns = not significant). NFL = No. flowers/inflorescence, FS = Fruit set, TNF = No. fruit/plant, FW = Fruit weight, YP = Yield/plant. Table S4: Pearson’s correlation index among the five traits evaluated in four experimental fields. In bold are reported values significant at least for *p* < 0.05 (* *p* < 0.05, ** *p* < 0.01, *** *p* < 0.001). C2016 = Campania in 2016, C2017 = Campania in 2017, P2016 = Puglia in 2016, P2017 = Puglia in 2017. NFL = No. flowers/inflorescence, FS = Fruit set, TNF = No. fruit/plant, FW = Fruit weight, YP = Yield/plant. Table S5: Significative Pearson’s correlation index evaluated between four traits (NFL, FS, FW, TNF) and production level (YP) for each genotype. In orange: negative correlation index, in green: positive correlation index. NFL = No. flowers/inflorescence, FS = Fruit set, TNF = No. fruit/plant, FW = Fruit weight, YP = Yield/plant, Table S6: Mean and stability parameters for yield per plant of 12 genotypes averaged over two locations (Campania and Puglia) and two years (2016-2017). Stability parameters: b_i_^v^, linear regression coefficient, ADL, average deviation from linearity, and *r^2^*, coefficient of determination. For ADL (Average Deviation from Linearity), * *p* < 0.05 with HSD Tukey’s test. Table S7: List of the 201 genes with a moderate impact effect on the protein structure. In bold, genes with private SNP variants for E42. For each gene, the position of the mutation in the tomato genome (version SL3.0), the genotypes carrying the mutation, the predicted effect, and the protein function are reported Table S8. Percentage of identity of 63 private InDels for E42 with three wild tomato species. Markers coded S are SSR markers and coded N are InDels. For each mutation, the percentage of identity and the position on scaffolds/contigs are reported for the related wild species’ genomes.
