# Supplementary material for: High-Throughput Genotyping of Resilient Tomato Landraces to Detect Candidate Genes Involved in the Response to High Temperatures
Source: Genes (Basel). 2020 Jun 7;11(6):626. doi: 10.3390/genes11060626 (PMC7349060; doi:10.3390/genes11060626)
Supplement: Supplementary file 1 [file genes-11-00626-s001.zip › Supplementary material/Supplementary Figure S1.pptx]

## Slide 1
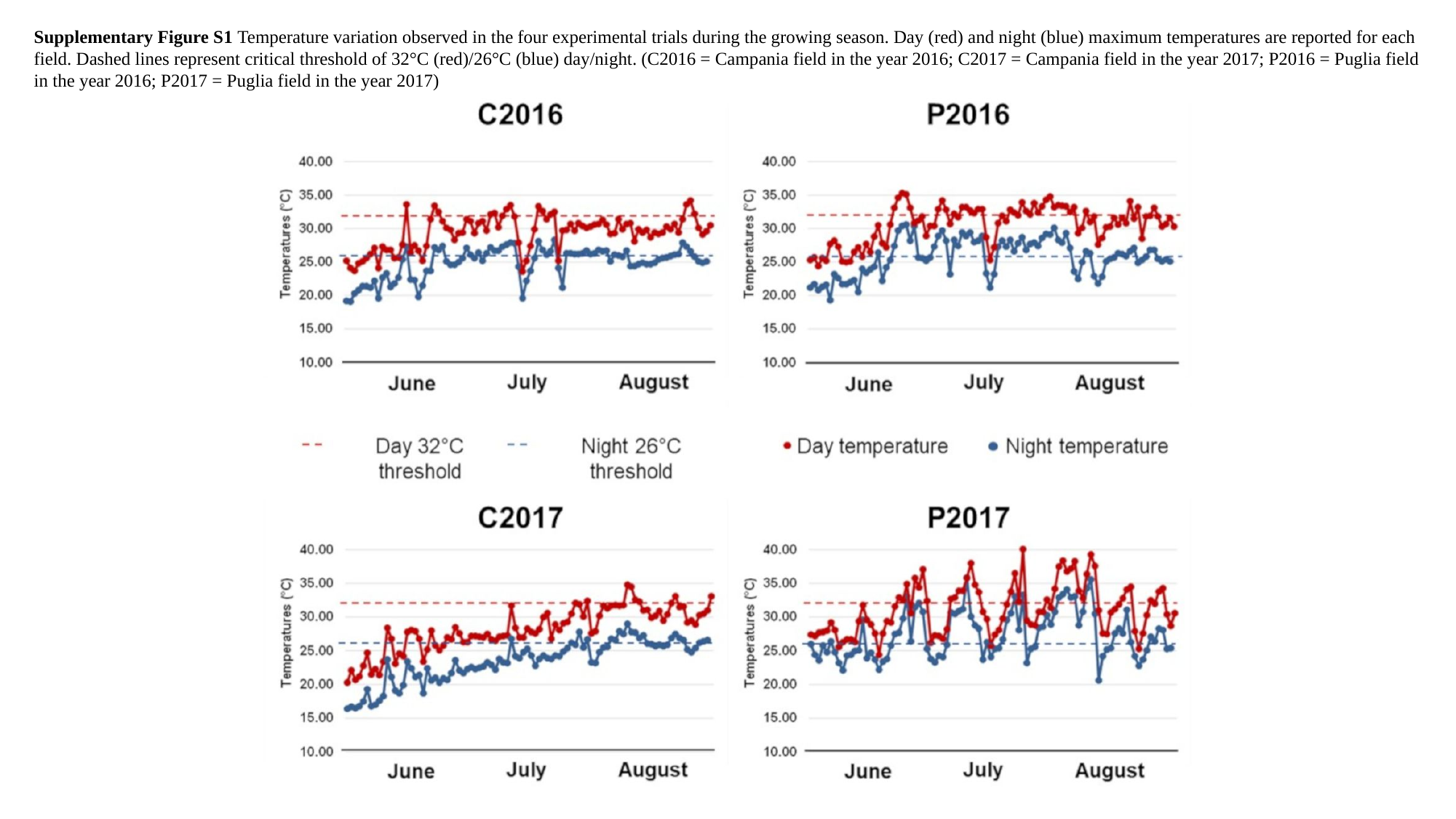

Supplementary Figure S1 Temperature variation observed in the four experimental trials during the growing season. Day (red) and night (blue) maximum temperatures are reported for each field. Dashed lines represent critical threshold of 32°C (red)/26°C (blue) day/night. (C2016 = Campania field in the year 2016; C2017 = Campania field in the year 2017; P2016 = Puglia field in the year 2016; P2017 = Puglia field in the year 2017)
