# Supplementary material for: High-Throughput Genotyping of Resilient Tomato Landraces to Detect Candidate Genes Involved in the Response to High Temperatures
Source: Genes (Basel). 2020 Jun 7;11(6):626. doi: 10.3390/genes11060626 (PMC7349060; doi:10.3390/genes11060626)
Supplement: Supplementary file 1 [file genes-11-00626-s001.zip › Supplementary material/Supplementary Figure S2.pptx]

## Slide 1
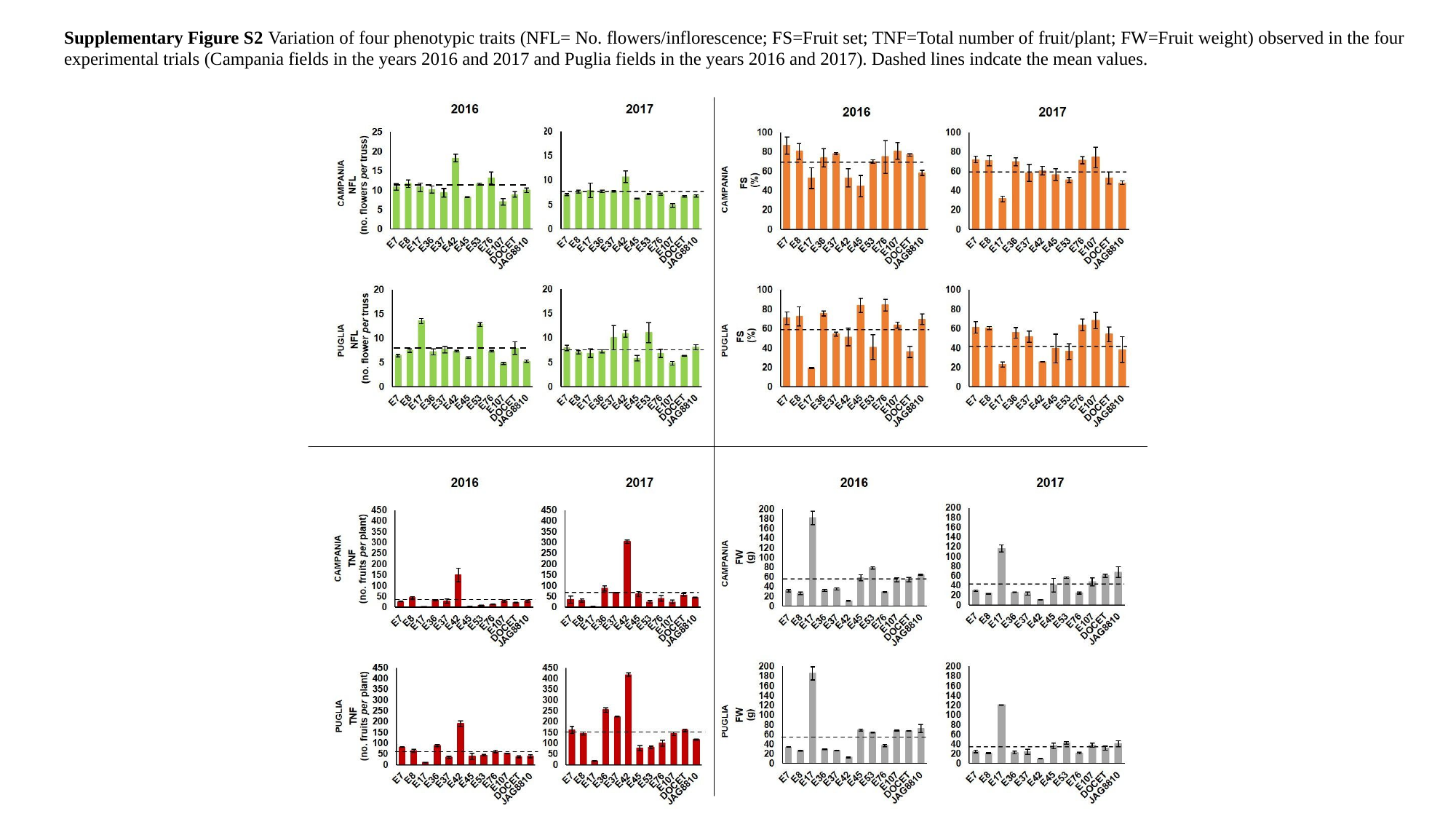

Supplementary Figure S2 Variation of four phenotypic traits (NFL= No. flowers/inflorescence; FS=Fruit set; TNF=Total number of fruit/plant; FW=Fruit weight) observed in the four experimental trials (Campania fields in the years 2016 and 2017 and Puglia fields in the years 2016 and 2017). Dashed lines indcate the mean values.
