# Supplementary material for: High-Throughput Genotyping of Resilient Tomato Landraces to Detect Candidate Genes Involved in the Response to High Temperatures
Source: Genes (Basel). 2020 Jun 7;11(6):626. doi: 10.3390/genes11060626 (PMC7349060; doi:10.3390/genes11060626)
Supplement: Supplementary file 1 [file genes-11-00626-s001.zip › Supplementary material/Supplementary Figure S3.pptx]

## Slide 1
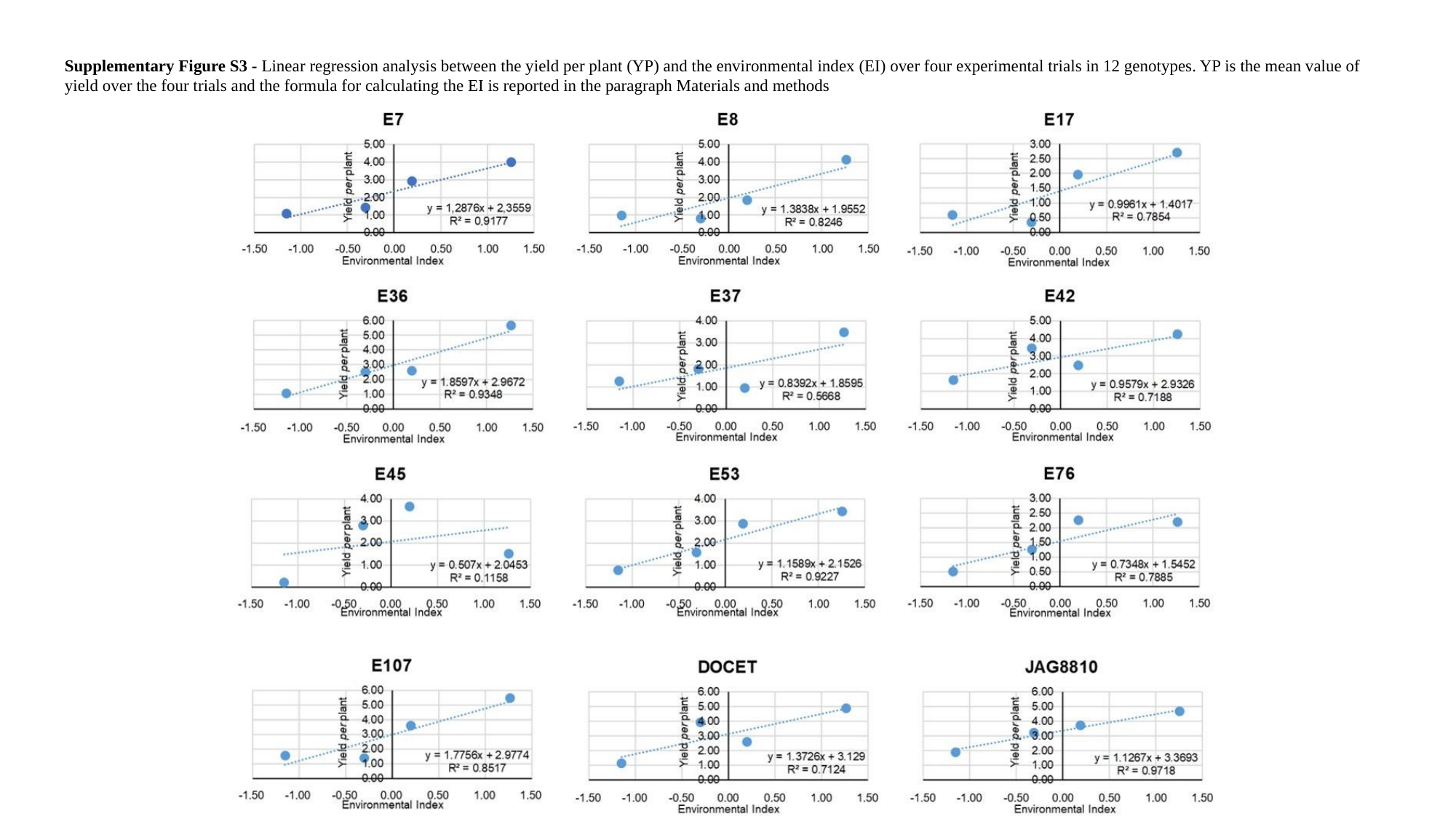

Supplementary Figure S3 - Linear regression analysis between the yield per plant (YP) and the environmental index (EI) over four experimental trials in 12 genotypes. YP is the mean value of yield over the four trials and the formula for calculating the EI is reported in the paragraph Materials and methods
