# Supplementary material for: High-Throughput Genotyping of Resilient Tomato Landraces to Detect Candidate Genes Involved in the Response to High Temperatures
Source: Genes (Basel). 2020 Jun 7;11(6):626. doi: 10.3390/genes11060626 (PMC7349060; doi:10.3390/genes11060626)

## Slide 1
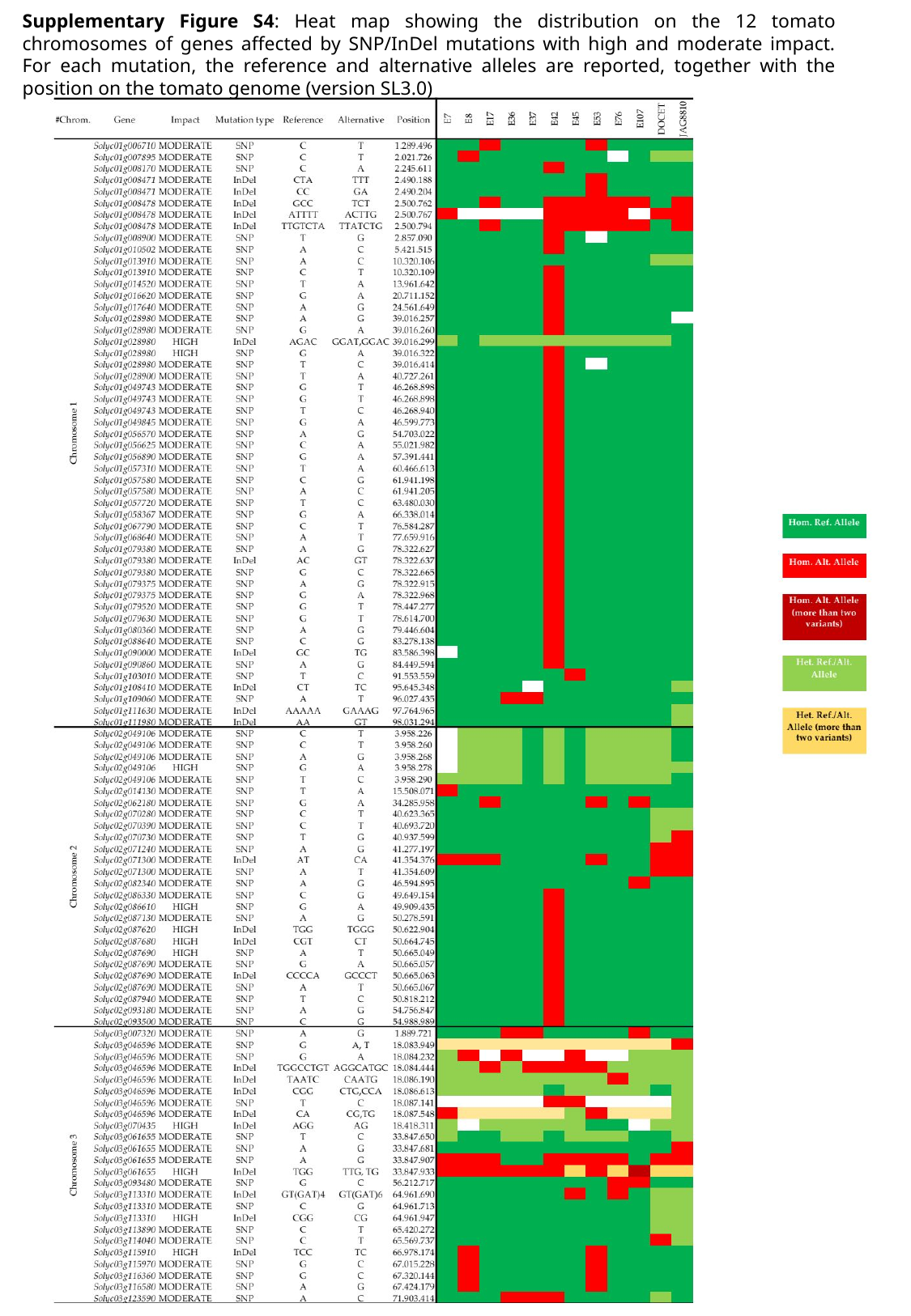

Supplementary Figure S4: Heat map showing the distribution on the 12 tomato chromosomes of genes affected by SNP/InDel mutations with high and moderate impact. For each mutation, the reference and alternative alleles are reported, together with the position on the tomato genome (version SL3.0)

## Slide 2
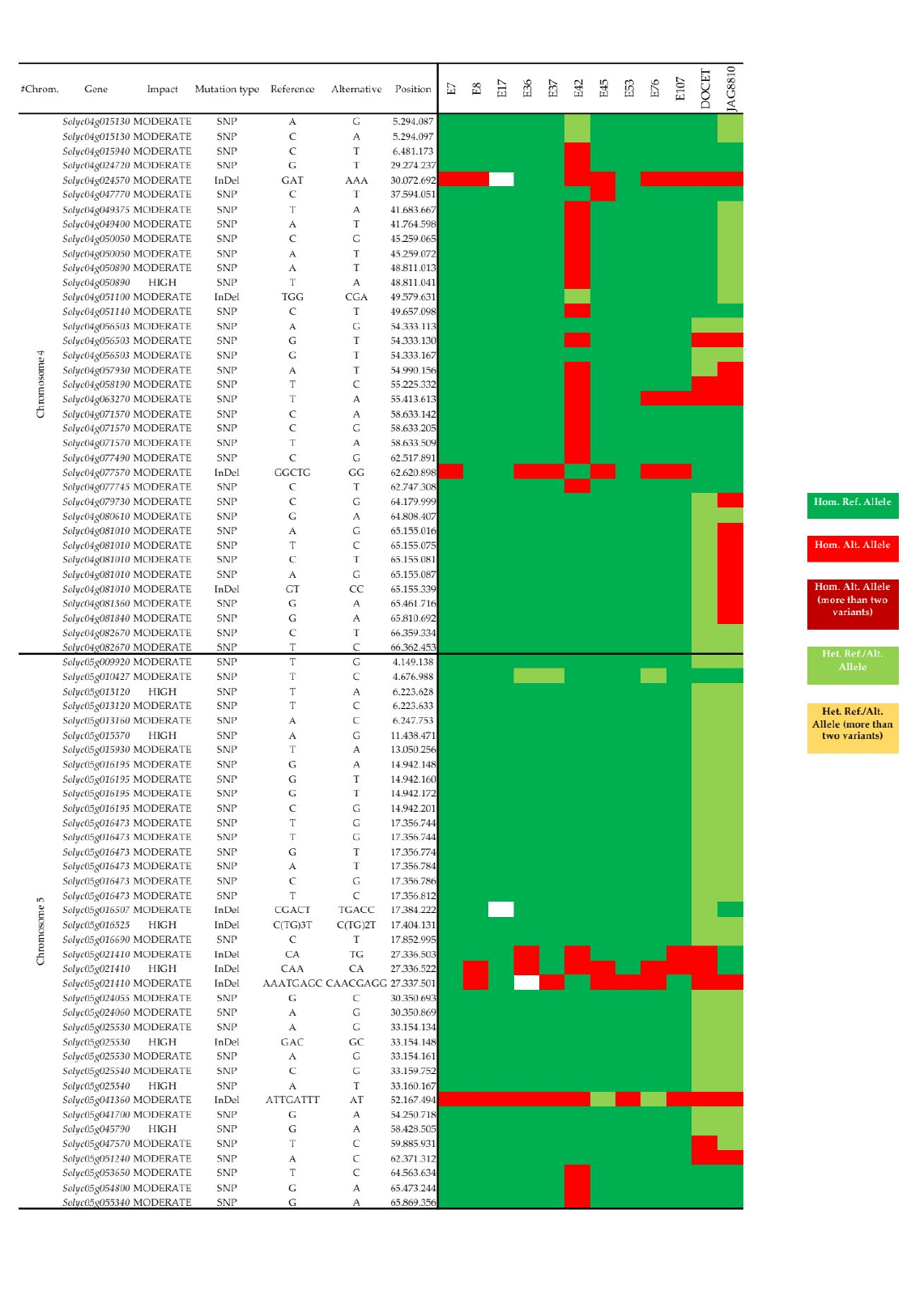

## Slide 3
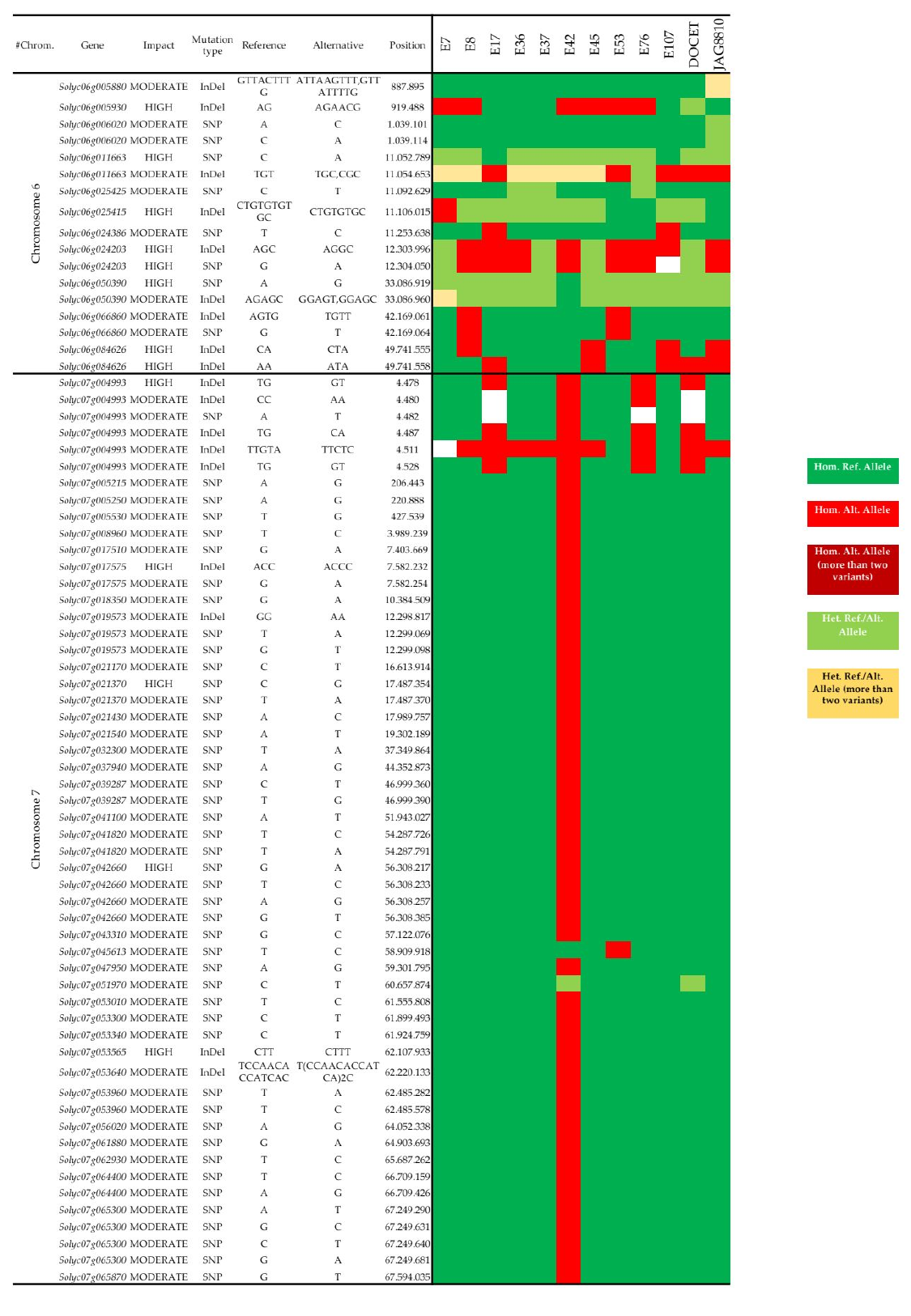

## Slide 4
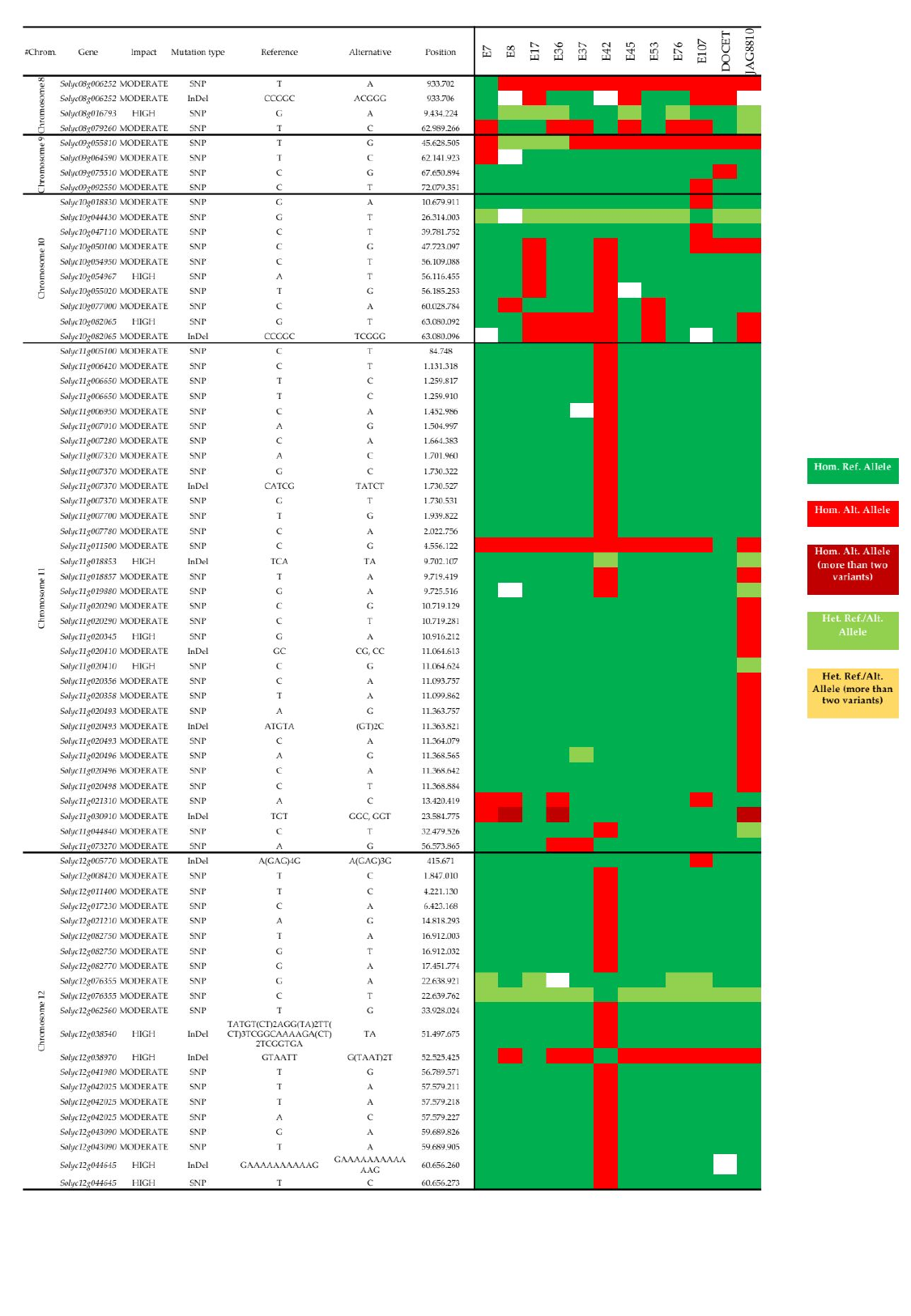

Supplement: Supplementary file 1 [file genes-11-00626-s001.zip › Supplementary material/Supplementary Figure S4.pptx]
