# Supplementary material for: High-Throughput Genotyping of Resilient Tomato Landraces to Detect Candidate Genes Involved in the Response to High Temperatures
Source: Genes (Basel). 2020 Jun 7;11(6):626. doi: 10.3390/genes11060626 (PMC7349060; doi:10.3390/genes11060626)
Supplement: Supplementary file 1 [file genes-11-00626-s001.zip › Supplementary material/Supplementary Table S1.docx]

**Supplementary Table S1**: List of the genotypes used for the phenotypic and genotypic screening. Genotypes coded E are ten tomato landraces collected at the University of Naples Federico II, and previously characterized. The genotypes DOCET and JAG8810 are hybrids kindly provided by Monsanto Italia. For each genotype, data are reported concerning their source, common name, country of origin, product destination, fruit size and shape

| **Genotype** | **Source** | **Common Name** | **Country of origin** | **Product destination** | **Fruit size** | **Fruit shape** |
| --- | --- | --- | --- | --- | --- | --- |
| E7 | CRA-ORT^1^ | Corbarino PC04 | Italy | Processing | Small (25-30 g) | Ovate |
| E8 | CRA-ORT^1^ | Corbarino PC05 | Italy | Processing | Small (20-25 g) | Elliptic |
| E17 | CRA-ORT^1^ | Pantano Romanesco | Italy | Fresh market | Big (200-250 g) | Flattened |
| E36 | Campania Region^1^ | Vesuvio Foglia Riccia | Italy | Fresh market/processing | Small (25-30 g) | Ovate |
| E37 | UNINA | Siccagno | Italy | Fresh market | Small (25-30 g) | Ovate |
| E42 | UNINA | PI15250 | Italy | Fresh market/processing | Small (25-30 g) | Circular |
| E45 | ARCA2010^1^ | SM246 | Italy | Processing | Medium (80-100 g) | Cylindric |
| E53 | TGRC | - | Honduras | Fresh market | Medium (80-100 g) | Oblate |
| E76 | TGRC | Black Plum | URSS | Processing | Small (20-25 g) | Ovate |
| E107 | NPGS | E-L-19 | Spain | Fresh market | Medium (80-100 g) | Round |
| DOCET | Monsanto Italia | DOCET | Italy | Processing | Medium (80-100 g) | Cylindric |
| JAG8810 | Monsanto Italia | JAG8810 | Italy | Processing | Medium (80-100 g) | Cylindric |

^1^ Germplasm collections maintained at public Italian Institutions
