# Supplementary material for: High-Throughput Genotyping of Resilient Tomato Landraces to Detect Candidate Genes Involved in the Response to High Temperatures
Source: Genes (Basel). 2020 Jun 7;11(6):626. doi: 10.3390/genes11060626 (PMC7349060; doi:10.3390/genes11060626)
Supplement: Supplementary file 1 [file genes-11-00626-s001.zip › Supplementary material/Supplementary Table S2.docx]

**Supplementary Table S2** Descriptive statistics of five traits evaluated on ten landraces (code E) and two control hybrids DOCET and JAG8810. Data recorded in: A) Campania year 2016; B) Campania year 2017; C) Puglia year 2016; D) Puglia year 2017. NFL=No. flowers/inflorescence; FS=Fruit set; TNF=Total number of fruit/plant; FW=Fruit weight; YP=Yield/plant.

| CAMPANIA 2016 | NFL | | FS | | TNF | | FW | | YP | |
| --- | --- | --- | --- | --- | --- | --- | --- | --- | --- | --- |
|  | (No.) | | (%) | | (No.) | | (g) | | (Kg) | |
| Genotype | Mean | SE | Mean | SE | Mean | SE | Mean | SE | Mean | SE |
| E7 | 10.91 | 0.86 | 86.38 | 8.81 | 27.56 | 1.26 | 31.71 | 3.01 | 1.09 | 0.25 |
| E8 | 11.72 | 0.96 | 80.36 | 8.01 | 44.52 | 5.62 | 26.45 | 2.95 | 0.99 | 0.16 |
| E17 | 10.82 | 1.09 | 52.95 | 10.59 | 3.23 | 0.02 | 181.81 | 13.81 | 0.59 | 0.04 |
| E36 | 10.25 | 0.90 | 73.81 | 9.46 | 33.19 | 3.07 | 32.05 | 2.35 | 1.07 | 0.03 |
| E37 | 9.38 | 1.13 | 78.10 | 1.00 | 28.62 | 9.59 | 35.28 | 2.01 | 1.25 | 0.13 |
| E42 | 18.33 | 1.02 | 53.08 | 9.49 | 149.99 | 31.21 | 10.78 | 0.30 | 1.61 | 0.29 |
| E45 | 8.24 | 0.10 | 44.76 | 10.79 | 3.90 | 0.90 | 58.21 | 5.77 | 0.38 | 0.17 |
| E53 | 11.64 | 0.28 | 69.83 | 1.74 | 6.95 | 2.54 | 78.89 | 2.51 | 0.76 | 0.08 |
| E76 | 13.19 | 1.56 | 74.70 | 16.72 | 13.48 | 0.27 | 29.52 | 1.01 | 0.50 | 0.11 |
| E107 | 7.03 | 0.81 | 80.81 | 8.60 | 28.83 | 5.72 | 53.91 | 4.11 | 1.53 | 0.19 |
| DOCET | 8.96 | 0.67 | 76.75 | 1.40 | 20.56 | 2.34 | 54.73 | 4.93 | 1.11 | 0.03 |
| JAG8810 | 10.06 | 0.57 | 58.08 | 2.53 | 29.28 | 4.06 | 64.60 | 1.32 | 1.89 | 0.22 |
| Descriptive statistics | | | | | | | | | | |
| Min | 7.03 |  | 44.76 |  | 3.23 |  | 10.78 |  | 0.38 |  |
| Max | 18.33 |  | 86.38 |  | 149.99 |  | 181.81 |  | 1.89 |  |
| Mean | 10.88 |  | 69.13 |  | 32.51 |  | 54.82 |  | 1.06 |  |
| SE | 0.83 |  | 3.88 |  | 11.30 |  | 12.80 |  | 0.13 |  |

| CAMPANIA 2017 | NFL | | FS | | TNF | | FW | | YP | |
| --- | --- | --- | --- | --- | --- | --- | --- | --- | --- | --- |
|  | (No.) | | (%) | | (No.) | | (g) | | (Kg) | |
| Genotype | Mean | SE | Mean | SE | Mean | SE | Mean | SE | Mean | SE |
| E7 | 7.05 | 0.20 | 71.90 | 3.50 | 35.84 | 16.06 | 29.51 | 1.38 | 1.42 | 0.77 |
| E8 | 7.67 | 0.33 | 70.61 | 5.06 | 30.30 | 6.73 | 23.49 | 0.09 | 0.82 | 0.19 |
| E17 | 7.91 | 1.55 | 31.52 | 2.93 | 3.05 | 2.25 | 116.28 | 6.85 | 0.34 | 0.24 |
| E36 | 7.69 | 0.25 | 69.65 | 3.99 | 87.31 | 10.93 | 26.07 | 0.84 | 2.52 | 0.12 |
| E37 | 7.72 | 0.19 | 58.13 | 8.76 | 68.07 | 0.79 | 23.45 | 2.91 | 1.80 | 0.04 |
| E42 | 10.72 | 1.21 | 60.45 | 4.53 | 304.18 | 8.07 | 11.27 | 0.07 | 3.43 | 0.07 |
| E45 | 6.22 | 0.12 | 56.35 | 5.84 | 62.27 | 11.92 | 40.95 | 13.69 | 2.79 | 0.44 |
| E53 | 7.14 | 0.12 | 50.97 | 2.37 | 24.70 | 5.31 | 56.68 | 1.43 | 1.56 | 0.46 |
| E76 | 7.17 | 0.22 | 71.24 | 3.82 | 41.33 | 13.42 | 24.92 | 2.07 | 1.25 | 0.38 |
| E107 | 4.82 | 0.36 | 74.23 | 10.77 | 24.50 | 8.23 | 47.75 | 8.20 | 1.35 | 0.09 |
| DOCET | 6.67 | 0.17 | 53.07 | 6.08 | 57.40 | 6.21 | 60.13 | 3.01 | 3.93 | 0.58 |
| JAG8810 | 6.80 | 0.27 | 48.08 | 1.67 | 44.90 | 2.78 | 67.80 | 11.01 | 3.23 | 0.33 |
| Descriptive statistics | | | | | | | | | | |
| Min | 4.82 |  | 31.52 |  | 3.05 |  | 11.27 |  | 0.34 |  |
| Max | 10.72 |  | 74.23 |  | 304.18 |  | 116.28 |  | 3.93 |  |
| Mean | 7.23 |  | 59.68 |  | 65.32 |  | 44.03 |  | 2.04 |  |
| SE | 0.39 |  | 3.67 |  | 22.68 |  | 8.28 |  | 0.32 |  |

| PUGLIA 2016 | NFL | | FS | | TNF | | FW | | YP | |
| --- | --- | --- | --- | --- | --- | --- | --- | --- | --- | --- |
|  | (No.) | | (%) | | (No.) | | (g) | | (Kg) | |
| Genotype | Mean | SE | Mean | SE | Mean | SE | Mean | SE | Mean | SE |
| E7 | 6.41 | 0.23 | 70.84 | 6.51 | 82.10 | 1.35 | 34.33 | 0.46 | 2.93 | 0.17 |
| E8 | 7.45 | 0.39 | 72.70 | 10.01 | 65.63 | 6.79 | 26.55 | 0.70 | 1.86 | 0.03 |
| E17 | 13.50 | 0.50 | 19.62 | 0.38 | 10.67 | 1.20 | 185.94 | 13.3 | 1.96 | 0.17 |
| E36 | 7.22 | 0.61 | 75.62 | 2.59 | 88.48 | 4.55 | 29.48 | 0.98 | 2.60 | 0.13 |
| E37 | 7.66 | 0.68 | 54.15 | 2.09 | 34.83 | 4.60 | 26.79 | 0.65 | 0.94 | 0.15 |
| E42 | 7.33 | 0.19 | 51.40 | 9.06 | 190.49 | 12.01 | 12.97 | 1.07 | 2.47 | 0.28 |
| E45 | 6.00 | 0.19 | 83.95 | 7.43 | 39.82 | 14.39 | 68.89 | 2.11 | 3.65 | 0.74 |
| E53 | 12.78 | 0.43 | 40.90 | 12.81 | 44.67 | 3.67 | 64.24 | 1.03 | 2.87 | 0.28 |
| E76 | 7.33 | 0.19 | 84.35 | 5.85 | 61.52 | 5.86 | 36.92 | 2.17 | 2.25 | 0.11 |
| E107 | 4.78 | 0.22 | 63.49 | 3.11 | 52.53 | 1.69 | 68.07 | 0.88 | 3.58 | 0.16 |
| DOCET | 7.94 | 1.29 | 36.40 | 5.72 | 36.93 | 5.64 | 67.62 | 0.19 | 2.60 | 0.29 |
| JAG8810 | 5.25 | 0.25 | 69.62 | 5.42 | 39.40 | 6.71 | 72.63 | 8.18 | 3.72 | 0.31 |
| Descriptive statistics | | | | | | | | | | |
| Min | 4.78 |  | 19.62 |  | 10.67 |  | 12.97 |  | 0.94 |  |
| Max | 13.5 |  | 84.35 |  | 190.49 |  | 185.94 |  | 3.72 |  |
| Mean | 7.80 |  | 60.25 |  | 62.25 |  | 57.87 |  | 2.62 |  |
| SE | 0.77 |  | 5.82 |  | 13.22 |  | 13.13 |  | 0.24 |  |

| PUGLIA 2017 | NFL | | FS | | TNF | | FW | | YP | |
| --- | --- | --- | --- | --- | --- | --- | --- | --- | --- | --- |
|  | (No.) | | (%) | | (No.) | | (g) | | (Kg) | |
| Genotype | Mean | SE | Mean | SE | Mean | SE | Mean | SE | Mean | SE |
| E7 | 7.94 | 0.56 | 61.21 | 5.94 | 161.53 | 13.04 | 24.68 | 2.68 | 3.99 | 0.57 |
| E8 | 7.03 | 0.27 | 60.60 | 1.80 | 145.37 | 55.6 | 21.63 | 0.91 | 4.15 | 0.31 |
| E17 | 6.86 | 0.90 | 23.17 | 2.62 | 19.16 | 5.50 | 121.19 | 0.48 | 2.72 | 0.29 |
| E36 | 7.22 | 0.29 | 55.72 | 5.48 | 254.27 | 39.78 | 22.82 | 2.24 | 5.67 | 0.58 |
| E37 | 10.06 | 2.48 | 51.56 | 5.70 | 222.87 | 61.65 | 24.62 | 5.18 | 3.45 | 0.42 |
| E42 | 10.92 | 0.72 | 25.84 | 0.29 | 417.25 | 47.32 | 10.20 | 0.67 | 4.22 | 0.39 |
| E45 | 5.83 | 0.58 | 39.54 | 14.97 | 76.00 | 23.24 | 36.70 | 5.75 | 1.53 | 0.16 |
| E53 | 11.11 | 2.10 | 36.45 | 7.86 | 80.37 | 2.65 | 42.50 | 2.57 | 3.42 | 0.25 |
| E76 | 6.86 | 0.82 | 63.98 | 6.10 | 100.47 | 19.37 | 21.79 | 1.66 | 2.18 | 0.41 |
| E107 | 4.83 | 0.36 | 68.50 | 8.30 | 143.92 | 6.48 | 37.71 | 4.32 | 5.45 | 0.87 |
| DOCET | 6.28 | 0.12 | 54.14 | 7.39 | 159.96 | 24.57 | 31.41 | 4.09 | 4.88 | 0.57 |
| JAG8810 | 8.07 | 0.49 | 38.30 | 13.27 | 116.93 | 16.97 | 41.41 | 6.33 | 4.65 | 0.38 |
| Descriptive statistics | | | | | | | | | | |
| Min | 4.83 |  | 23.17 |  | 19.16 |  | 10.20 |  | 1.53 |  |
| Max | 11.11 |  | 68.50 |  | 417.25 |  | 121.19 |  | 5.67 |  |
| Mean | 7.75 |  | 48.25 |  | 158.17 |  | 36.39 |  | 3.86 |  |
| SE | 0.57 |  | 4.37 |  | 29.91 |  | 8.19 |  | 0.37 |  |
