# Supplementary material for: High-Throughput Genotyping of Resilient Tomato Landraces to Detect Candidate Genes Involved in the Response to High Temperatures
Source: Genes (Basel). 2020 Jun 7;11(6):626. doi: 10.3390/genes11060626 (PMC7349060; doi:10.3390/genes11060626)
Supplement: Supplementary file 1 [file genes-11-00626-s001.zip › Supplementary material/Supplementary Table S3.docx]

**Supplementary Table S3** Three-way ANOVA for traits measured on 12 genotypes in two fields and two years. The level of significance (p) and the total sum of square percentage (TSS %) are reported. (*p<0.05, **p<0.01, ***p<0.001, ns not significant). NFL=No. flowers/inflorescence; FS=Fruit set; TNF=No. fruit/plant; FW=Fruit weight; YP=Yield/plant

| Source of variation | NFL | | FS | | TNF | | FW | | YP | |
| --- | --- | --- | --- | --- | --- | --- | --- | --- | --- | --- |
|  | TSS % | Sign. | TSS % | Sign. | TSS % | Sign. | TSS % | Sign. | TSS % | Sign. |
| Genotype (G) | 31.16 | *** | 40.20 | *** | 47.63 | *** | 84.50 | *** | 19.72 | *** |
| Location (L) | 6.96 | *** | 7.44 | *** | 13.05 | *** | 0.08 | ns | 35.01 | *** |
| Year (Y) | 10.23 | *** | 7.38 | *** | 14.10 | *** | 5.01 | *** | 15.09 | *** |
| Genotype x Location (G x L) | 9.00 | *** | 6.99 | * | 2.52 | * | 0.64 | ns | 5.38 | *** |
| Genotype x Year (G x Y) | 3.58 | ns | 2.18 | ns | 7.96 | *** | 4.60 | *** | 6.79 | *** |
| Location x Year (L x Y) | 11.45 | *** | 0.06 | ns | 3.35 | *** | 0.61 | *** | 0.20 | ns |
| Genotype x Location x Year (G x L x Y) | 8.42 | *** | 12.07 | *** | 1.95 | ns | 1.10 | * | 9.56 | *** |
| Error | 19.20 |  | 23.68 |  | 9.44 |  | 3.46 |  | 8.28 |  |
