# Supplementary material for: High-Throughput Genotyping of Resilient Tomato Landraces to Detect Candidate Genes Involved in the Response to High Temperatures
Source: Genes (Basel). 2020 Jun 7;11(6):626. doi: 10.3390/genes11060626 (PMC7349060; doi:10.3390/genes11060626)
Supplement: Supplementary file 1 [file genes-11-00626-s001.zip › Supplementary material/Supplementary Table S4.docx]

**Supplementary Table S4** Pearson’s correlation index among the five traits evaluated in four experimental fields. In bold are reported the values significant at least for p<0.05 (*p<0.05; **p<0.01; ***p<0.001). C2016=Campania in 2016; C2017=Campania in 2017; P2016=Puglia in 2016; P2017=Puglia in 2017. NFL=No. flowers/inflorescence; FS=Fruit set; TNF=No. fruit/plant; FW=Fruit weight; YP=Yield/plant.

| Field | Trait | NFL | FS | TNF | FW | YP |
| --- | --- | --- | --- | --- | --- | --- |
| C2016 | NFL |  |  |  |  |  |
| C2017 |  |  |  |  |  |  |
| P2016 |  |  |  |  |  |  |
| P2017 |  |  |  |  |  |  |
| C2016 | FS | -0.14 |  |  |  |  |
| C2017 |  | -0.12 |  |  |  |  |
| P2016 |  | **-0.57^***^** |  |  |  |  |
| P2017 |  | **-0.39^*^** |  |  |  |  |
| C2016 | TNF | **0.60^***^** | 0.13 |  |  |  |
| C2017 |  | **0.46^**^** | 0.08 |  |  |  |
| P2016 |  | -0.12 | 0.12 |  |  |  |
| P2017 |  | **0.40^*^** | -0.11 |  |  |  |
| C2016 | FW | -0.26 | **-0.49^*^** | **-0.45^*^** |  |  |
| C2017 |  | -0.03 | **-0.63^***^** | **-0.45^**^** |  |  |
| P2016 |  | **0.47^**^** | **-0.52^**^** | **-0.63^***^** |  |  |
| P2017 |  | -0.16 | -0.25 | **-0.59^***^** |  |  |
| C2016 | YP | 0.14 | 0.26 | **0.56^**^** | -0.28 |  |
| C2017 |  | -0.09 | 0.02 | **0.53^**^** | -0.11 |  |
| P2016 |  | **-0.42^*^** | 0.19 | 0.11 | -0.03 |  |
| P2017 |  | 0.01 | 0.07 | **0.51^**^** | -0.17 |  |
