# Supplementary material for: High-Throughput Genotyping of Resilient Tomato Landraces to Detect Candidate Genes Involved in the Response to High Temperatures
Source: Genes (Basel). 2020 Jun 7;11(6):626. doi: 10.3390/genes11060626 (PMC7349060; doi:10.3390/genes11060626)
Supplement: Supplementary file 1 [file genes-11-00626-s001.zip › Supplementary material/Supplementary Table S5.docx]

**Supplementary Table S5** Significative Pearson’s correlation index evaluated between four traits (NFL, FS, FW, TNF) and production level (YP) for each genotype. In orange: negative correlation index; in green: positive correlation index. NFL = No. flowers/inflorescence; FS=Fruit set; TNF=No. fruit/plant; FW=Fruit weight; YP=Yield/plant.

| Genotype | NFL | FS | FW | TNF |
| --- | --- | --- | --- | --- |
| E7 |  |  |  | 0.00 |
| E8 |  |  |  | 0.00 |
| E17 |  |  |  | 0.00 |
| E36 | 0.03 | 0.02 | 0.02 | 0.00 |
| E37 |  |  |  | 0.00 |
| E42 |  | 0.04 |  | 0.00 |
| E45 |  |  |  | 0.00 |
| E53 |  | 0.02 | 0.01 | 0.00 |
| E76 |  |  |  | 0.00 |
| E107 |  |  |  | 0.00 |
| DOCET | 0.01 |  |  | 0.01 |
| JAG8810 |  |  |  | 0.01 |
