# Supplementary material for: High-Throughput Genotyping of Resilient Tomato Landraces to Detect Candidate Genes Involved in the Response to High Temperatures
Source: Genes (Basel). 2020 Jun 7;11(6):626. doi: 10.3390/genes11060626 (PMC7349060; doi:10.3390/genes11060626)
Supplement: Supplementary file 1 [file genes-11-00626-s001.zip › Supplementary material/Supplementary Table S6.docx]

**Supplementary Table S6** Mean and stability parameters for yield per plant of 12 genotypes averaged over two locations (Campania and Puglia) and two years (2016-2017). Stability parameters: b_i_^v^, linear regression coefficient; ADL, average deviation from linearity; r^2^, coefficient of determination. For ADL, *p<0.05 with HSD Tukey’s test.

| **Genotype** | **Yield *per* plant**  **(Kg)** | **Stability parameters** | | |
| --- | --- | --- | --- | --- |
|  |  | **b_i_^v^** | **ADL** | **r^2^** |
| E7 | 2.36 | 1.29 | 0.00 | 0.92 |
| E8 | 1.96 | 1.38 | 0.15* | 0.82 |
| E17 | 1.40 | 1.00 | 0.02 | 0.79 |
| E36 | 2.97 | 1.86 | 0.03 | 0.93 |
| E37 | 1.86 | 0.84 | 0.12* | 0.57 |
| E42 | 2.93 | 0.96 | 0.00 | 0.72 |
| E45 | 2.05 | 0.51 | 0.72* | 0.12 |
| E53 | 2.15 | 1.16 | 0.01 | 0.92 |
| E76 | 1.55 | 0.73 | 0.04 | 0.79 |
| E107 | 2.98 | 1.78 | 0.05 | 0.85 |
| DOCET | 3.13 | 1.37 | 0.01 | 0.71 |
| JAG8810 | 3.37 | 1.13 | 0.01 | 0.97 |
