# Supplementary material for: High-Throughput Genotyping of Resilient Tomato Landraces to Detect Candidate Genes Involved in the Response to High Temperatures
Source: Genes (Basel). 2020 Jun 7;11(6):626. doi: 10.3390/genes11060626 (PMC7349060; doi:10.3390/genes11060626)
Supplement: Supplementary file 1 [file genes-11-00626-s001.zip › Supplementary material/Supplementary Table S7.docx]

**Supplementary Table S7** List of the 201 genes showing mutations with a moderate impact effect on the protein structure. For each gene, the position of the mutation in the tomato genome (version SL3.0), the genotypes carrying the mutation, the predicted effect and the protein function are reported.

| **Gene** | **Mutation type** | **Position** | **Mutated genotypes** | **Predicted effect** | **Protein function** |
| --- | --- | --- | --- | --- | --- |
| *Solyc01g006710* | SNP | 1,289,496 | E17, E53 | missense variant | ATP-dependent helicase |
| *Solyc01g007895* | SNP | 2,021,726 | E8, DOCET, JAG8810 | missense variant &  splice region variant | Basic helix-loop-helix (bHLH) DNA-binding superfamily protein |
| *Solyc01g008170* | SNP | 2,245,611 | E42 | missense variant | Zinc finger transcription factor 5 |
| *Solyc01g008471* | InDel | 2,490,188 | E53 | missense variant | Histone-lysine N-methyltransferase SUVR5 |
|  | InDel | 2,490,204 | E53 | missense variant |  |
| *Solyc01g008478* | InDel | 2,500,762 | E17, E42, E45, E53, E76, E107, JAG8810 | missense variant | Histone-lysine N-methyltransferase SUVR5 |
|  | InDel | 2,500,767 | E7, E42, E45, E53, E76, DOCET, JAG8810 | missense variant |  |
|  | InDel | 2,500,794 | E17, E42, E45, E53, E76, E07, JAG8810 | missense variant |  |
| *Solyc01g008900* | SNP | 2,857,090 | E42 | missense variant | Pyridoxal-5'-phosphate-dependent enzyme family protein |
| *Solyc01g010502* | SNP | 5,421,515 | E42 | missense variant | F-box family protein |
| *Solyc01g013910* | SNP | 10,320,106 | DOCET, JAG8810 | missense variant &  splice region variant | Endoribonuclease Dicer 2b |
|  | SNP | 10,320,109 | E42 | missense variant |  |
| *Solyc01g014520* | SNP | 13,961,642 | E42 | missense variant | Serine/threonine-protein kinase |
| *Solyc01g016620* | SNP | 20,711,152 | E42 | missense variant | Oxidoreductase/transition metal ion-binding protein |
| *Solyc01g017640* | SNP | 24,561,649 | E42 | missense variant &  splice region variant | Leucine-rich repeat-containing protein 2 |
| *Solyc01g028980* | SNP | 39,016,257 | E42 | missense variant | Gamma-tubulin complex component |
|  | SNP | 39,016,260 | E42 | missense variant |  |
|  | SNP | 39,016,414 | E42 | missense variant |  |
| *Solyc01g028900* | SNP | 40,727,261 | E42 | missense variant | Pyruvate dehydrogenase E1 component subunit beta |
| *Solyc01g049743* | SNP | 46,268,898 | E42 | missense variant | Protein Ycf2 |
|  | SNP | 46,268,940 | E42 | missense variant |  |
| *Solyc01g049845* | SNP | 46,599,773 | E42 | missense variant | DNA-directed RNA polymerase subunit beta |
| *Solyc01g056570* | SNP | 54,703,022 | E42 | missense variant | Ankyrin repeat family protein / regulator of chromosome condensation (RCC1) family protein |
| *Solyc01g056625* | SNP | 55,021,982 | E42 | missense variant | Double-stranded RNA-binding |
| *Solyc01g056890* | InDel | 57,391,441 | E42 | missense variant | F-box/RNI-like/FBD-like domains-containing protein |
| *Solyc01g057310* | SNP | 60,466,613 | E42 | missense variant | Kinesin-like protein KIF15 |
| *Solyc01g057580* | SNP | 61,941,198 | E42 | missense variant | Kinase |
|  | SNP | 61,941,205 | E42 | missense variant |  |
| *Solyc01g057720* | SNP | 63,480,030 | E42 | missense variant | AT hook motif-containing protein |
| *Solyc01g058367* | SNP | 66,338,014 | E42 | missense variant | PIF1 helicase |
| *Solyc01g067790* | SNP | 76,584,287 | E42 | missense variant | BRCT domain-containing DNA repair protein |
| *Solyc01g068640* | SNP | 77,659,916 | E42 | missense variant | aarF domain-containing protein kinase |
| *Solyc01g079380* | SNP | 78,322,627 | E42 | missense variant | GRAS family transcription factor |
|  | InDel | 78,322,637 | E42 | missense variant |  |
|  | SNP | 78,322,665 | E42 | missense variant |  |
| *Solyc01g079375* | SNP | 78,322,915 | E42 | missense variant | P-loop containing nucleoside triphosphate hydrolases superfamily protein |
|  | SNP | 78,322,968 | E42 | missense variant |  |
| *Solyc01g079520* | SNP | 78,447,277 | E42 | missense variant | DNA mismatch repair protein |
| *Solyc01g079630* | SNP | 78,614,700 | E42 | missense variant | Sucrose-phosphatase-related family protein |
| *Solyc01g080360* | SNP | 79,446,604 | E42 | missense variant | Clathrin adaptor complexes medium subunit family protein |
| *Solyc01g088640* | SNP | 83,278,138 | E42 | missense variant | RING/U-box superfamily protein |
| *Solyc01g090000* | InDel | 83,586,398 | E42 | missense variant | BAX inhibitor 1 |
| *Solyc01g090860* | SNP | 84,449,594 | E42 | missense variant | Nucleotidyltransferase family protein |
| *Solyc01g103010* | SNP | 91,553,559 | E45 | missense variant | Cullin-associated NEDD8-dissociated protein 1 |
| *Solyc01g108410* | InDel | 95,645,348 | JAG8810 | missense variant | Pentatricopeptide repeat-containing protein |
| *Solyc01g109060* | SNP | 96,027,435 | E42, E45 | missense variant | Pentatricopeptide repeat-containing protein |
| *Solyc01g111630* | InDel | 97,764,965 | JAG8810 | missense variant &  splice region variant | D-3-phosphoglycerate dehydrogenase |
| *Solyc01g111980* | InDel | 98,031,294 | JAG8810 | missense variant | Amino acid transporter |
| *Solyc02g049106* | SNP | 3,958,226 | E8, E17, E36, E42, E53, E76, E107, DOCET | missense variant | Leucine-rich repeat protein kinase family protein |
|  | SNP | 3,958,260 | E8, E17, E36, E42, E53, E76, E107, DOCET | missense variant |  |
|  | SNP | 3,958,268 | E8, E17, E36, E42, E53, E76, E107, DOCET | missense variant |  |
|  | SNP | 3,958,290 | E7, E8, E17, E36, E42, E53, E76, E107, DOCET | missense variant |  |
| *Solyc02g014130* | SNP | 15,508,071 | E7 | missense variant | Tudor/PWWP/MBT domain-containing protein |
| *Solyc02g062180* | SNP | 34,285,958 | E17, E53, E107 | missense variant | 2-oxoglutarate |
| *Solyc02g070280* | SNP | 40,623,365 | DOCET, JAG8810 | missense variant | Cationic amino acid transporter |
| *Solyc02g070390* | SNP | 40,693,720 | DOCET, JAG8810 | missense variant | Plastid transcriptionally active 12 |
| *Solyc02g070730* | SNP | 40,937,599 | DOCET, JAG8810 | missense variant | NBS-LRR resistance protein |
| *Solyc02g071240* | SNP | 41,277,197 | DOCET, JAG8810 | missense variant | Pre-mRNA-processing factor 39 |
| *Solyc02g071300* | InDel | 41,354,376 | E7, E8, E17, E53, DOCET, JAG8810 | missense variant | Pentatricopeptide repeat (PPR) superfamily protein |
|  | SNP | 41,354,609 | DOCET, JAG8810 | missense variant |  |
| *Solyc02g082340* | SNP | 46,594,895 | E107 | missense variant | RNA polymerase I specific transcription initiation factor RRN3 protein |
| *Solyc02g086330* | SNP | 49,649,154 | E42 | missense variant | LisH domain and HEAT repeat-containing protein KIAA1468-like protein |
| *Solyc02g087130* | SNP | 50,278,591 | E42 | missense variant | RNase H family protein |
| *Solyc02g087690* | SNP | 50,665,057 | E42 | missense variant | FACT complex subunit SSRP1 |
|  | InDel | 50,665,063 | E42 | missense variant |  |
|  | SNP | 50,665,067 | E42 | missense variant |  |
| *Solyc02g087940* | SNP | 50,818,212 | E42 | missense variant | Coiled-coil domain-containing protein 115 |
| *Solyc02g093180* | SNP | 54,756,847 | E42 | missense variant | HXXXD-type acyl-transferase family protein |
| *Solyc02g093500* | SNP | 54,988,989 | E42 | missense variant | GDSL esterase/lipase family |
| *Solyc03g007320* | SNP | 1,889,721 | E36, E37, E107 | missense variant &  splice region variant | Polypyrimidine tract-binding protein |
| *Solyc03g046596* | InDel | 18,083,114 | E7, E8, E36, E37, E53, E76, E107, JAG8810 | missense variant | BCL-2-associated athanogene 6 |
|  | SNP | 18,083,949 | JAG8810 | missense variant |  |
|  | SNP | 18,084,232 | E8, E36, E45 | missense variant |  |
|  | InDel | 18,084,444 | E17, E37, E42, E45, E53 | missense variant |  |
|  | InDel | 18,086,190 | E76 | missense variant &  splice region variant |  |
|  | InDel | 18,086,613 | E42, E76, DOCET | missense variant |  |
|  | SNP | 18,087,141 | E53, E76 | missense variant |  |
|  | InDel | 18,087,548 | E7, E45, E53, JAG8810 | missense variant |  |
| *Solyc03g061655* | SNP | 33,847,650 | E7, E36, E37, E45, E53, E76 | missense variant | Ribosomal protein S12 |
|  | SNP | 33,847,681 | E53 | missense variant |  |
|  | SNP | 33,847,907 | E7, E8, E17, E42, E45, E53, E76, DOCET, JAG8810 | missense variant |  |
| *Solyc03g093480* | SNP | 56,212,717 | E76, E107 | missense variant | U3 small nucleolar RNA-associated protein 10 and NUC211 domain-containing protein |
| *Solyc03g113310* | InDel | 64,961,690 | E45, E76, DOCET, JAG8810 | conservative inframe insertion | Pseudouridine synthase family protein |
|  | SNP | 64,961,713 | DOCET, JAG8810 | missense variant |  |
| *Solyc03g113890* | SNP | 65,420,272 | DOCET, JAG8810 | missense variant | Zinc finger family protein |
| *Solyc03g114040* | SNP | 65,569,737 | DOCET, JAG8810 | missense variant | Protein PRD1 |
| *Solyc03g115970* | SNP | 67,015,228 | E8, E53 | missense variant | Protein yippee-like |
| *Solyc03g116360* | SNP | 67,320,144 | E8, E53 | missense variant | Regulator of chromosome condensation (RCC1) family protein |
| *Solyc03g116580* | SNP | 67,424,179 | E8, E53 | missense variant | Pectin lyase-like superfamily protein |
| *Solyc03g123590* | SNP | 71,903,414 | DOCET, E36, E37, E42 | missense variant | Remorin family protein |
| *Solyc04g015130* | SNP | 5,294,087 | E42, JAG8810 | missense variant | Protein kinase |
|  | SNP | 5,294,097 | E42, JAG8810 | missense variant |  |
| *Solyc04g015940* | SNP | 6,481,173 | E42 | missense variant | ARM repeat superfamily protein |
| *Solyc04g024720* | SNP | 29,274,237 | E42 | missense variant | Exostosin family protein |
| *Solyc04g024570* | InDel | 30,072,692 | E7, E8, E42, E45, E76, E107, DOCET, JAG8810 | missense variant | NADH dehydrogenase subunit |
| *Solyc04g047770* | SNP | 37,594,051 | E45 | missense variant | VHS domain-containing protein |
| *Solyc04g049375* | SNP | 41,683,667 | E42, JAG8810 | missense variant | ATP-citrate lyase B-1 |
| *Solyc04g049400* | SNP | 41,764,598 | E42, JAG8810 | missense variant | Kinase family protein |
| *Solyc04g050050* | SNP | 45,259,065 | E42, JAG8810 | missense variant | SUN-like protein 13 |
|  | SNP | 45,259,072 | E42, JAG8810 | missense variant |  |
| *Solyc04g050890* | SNP | 48,811,013 | E42, JAG8810 | missense variant | Dna-directed rna polymerase subunit alpha |
| *Solyc04g051100* | InDel | 49,579,631 | E42, JAG8810 | missense variant | Trichome birefringence-like protein (DUF828) |
| *Solyc04g051140* | SNP | 49,657,098 | E42 | missense variant | Phospholipase D |
| *Solyc04g056503* | SNP | 54,333,113 | DOCET, JAG8810 | missense variant | Tubulin beta chain |
|  | SNP | 54,333,130 | E42, DOCET, JAG8810 | missense variant |  |
|  | SNP | 54,333,167 | DOCET, JAG8810 | missense variant &  splice region variant |  |
| *Solyc04g057930* | SNP | 54,990,156 | E42, DOCET, JAG8810 | missense variant | Kinase family protein |
| *Solyc04g058190* | SNP | 55,225,332 | E42, DOCET, JAG8810 | missense variant | PHD finger family protein |
| *Solyc04g063270* | SNP | 55,413,613 | E42, E76, E107, DOCET, JAG8810 | missense variant | Pentatricopeptide repeat-containing family protein |
| *Solyc04g071570* | SNP | 58,633,142 | E42 | missense variant | SET domain protein |
|  | SNP | 58,633,205 | E42 | missense variant |  |
|  | SNP | 58,633,509 | E42 | missense variant |  |
| *Solyc04g077490* | SNP | 62,517,891 | E42 | missense variant | AP2-like ethylene-responsive transcription factor |
| *Solyc04g077570* | InDel | 62,620,898 | E7, E36, E37, E45, E76, E107 | disruptive inframe deletion | Transmembrane protein |
| *Solyc04g077745* | SNP | 62,747,308 | E42 | missense variant | NADH-ubiquinone oxidoreductase-like protein |
| *Solyc04g079730* | SNP | 64,179,999 | DOCET, JAG8810 | missense variant | Allene oxide synthase |
| *Solyc04g080610* | SNP | 64,808,407 | DOCET, JAG8810 | missense variant | Ornithine carbamoyltransferase |
| *Solyc04g081010* | SNP | 65,155,016 | DOCET, JAG8810 | missense variant | Forkhead-associated (FHA) domain-containing protein |
|  | SNP | 65,155,075 | DOCET, JAG8810 | missense variant |  |
|  | SNP | 65,155,081 | DOCET, JAG8810 | missense variant |  |
|  | SNP | 65,155,087 | DOCET, JAG8810 | missense variant |  |
|  | InDel | 65,155,339 | DOCET, JAG8810 | missense variant |  |
| *Solyc04g081360* | SNP | 65,461,716 | DOCET, JAG8810 | missense variant | tRNA (Adenine-N1-)-methyltransferase non-catalytic subunit trm6 |
| *Solyc04g081840* | SNP | 65,810,301 | DOCET, JAG8810 | missense variant | UPF0496 protein |
|  | SNP | 65,810,692 | DOCET, JAG8810 | missense variant |  |
| *Solyc04g082670* | SNP | 66,359,334 | DOCET, JAG8810 | missense variant | Dolichyl-diphosphooligosaccharide--protein glycosyltransferase subunit 1 |
|  | SNP | 66,362,453 | DOCET, JAG8810 | missense variant |  |
| *Solyc05g009920* | SNP | 4,149,138 | DOCET, JAG8810 | missense variant | P-loop containing nucleoside triphosphate hydrolases superfamily protein |
| *Solyc05g010427* | SNP | 4,676,988 | E36, E37, E76 | missense variant | Aminoalcoholphosphotransferase |
| *Solyc05g013120* | SNP | 6,223,633 | DOCET, JAG8810 | missense variant | Ninja-family protein AFP1 |
| *Solyc05g013160* | SNP | 6,247,753 | DOCET, JAG8810 | missense variant | Ribulose-1 |
| *Solyc05g015930* | SNP | 13,050,256 | DOCET, JAG8810 | missense variant | GYF domain-containing protein |
| *Solyc05g016195* | SNP | 14,942,148 | DOCET, JAG8810 | missense variant | Dead box ATP-dependent RNA helicase |
|  | SNP | 14,942,160 | DOCET, JAG8810 | missense variant |  |
|  | SNP | 14,942,172 | DOCET, JAG8810 | missense variant |  |
|  | SNP | 14,942,201 | DOCET, JAG8810 | missense variant |  |
| *Solyc05g016473* | SNP | 17,356,744 | DOCET, JAG8810 | missense variant | Pentatricopeptide repeat (PPR) superfamily protein |
|  | SNP | 17,356,774 | DOCET, JAG8810 | missense variant |  |
|  | SNP | 17,356,784 | DOCET, JAG8810 | missense variant |  |
|  | SNP | 17,356,786 | DOCET, JAG8810 | missense variant |  |
|  | SNP | 17,356,812 | DOCET, JAG8810 | missense variant |  |
| *Solyc05g016507* | InDel | 17,384,222 | DOCET | missense variant | GDSL-like lipase/acylhydrolase |
| *Solyc05g016690* | SNP | 17,852,995 | DOCET, JAG8810 | missense variant | Nudix hydrolase |
| *Solyc05g021410* | InDel | 27,336,503 | E36, E42, E107, DOCET | missense variant | histone-lysine N-methyltransferase SUVR5 |
|  | InDel | 27,337,501 | E8, E37, E45, E53, E76, DOCET, JAG8810 | missense variant |  |
| *Solyc05g024055* | SNP | 30,350,693 | DOCET, JAG8810 | missense variant | Lysine-specific histone demethylase 1-like protein |
| *Solyc05g024060* | SNP | 30,350,869 | DOCET, JAG8810 | missense variant | Lysine-specific histone demethylase 1-like protein |
| *Solyc05g025530* | SNP | 33,154,134 | DOCET, JAG8810 | missense variant | DNA-directed RNA polymerase subunit beta |
|  | SNP | 33,154,161 | DOCET, JAG8810 | missense variant |  |
| *Solyc05g025540* | SNP | 33,159,752 | DOCET, JAG8810 | missense variant | Molybdenum cofactor sulfurase |
| *Solyc05g041360* | InDel | 52,167,494 | E7, E8, E17, E36, E37, E42, E53, E107, DOCET, JAG8810 | disruptive inframe deletion | Maturase K |
| *Solyc05g041700* | SNP | 54,250,718 | DOCET, JAG8810 | missense variant | Auxin efflux carrier component |
| *Solyc05g047570* | SNP | 59,885,931 | DOCET, JAG8810 | missense variant | SLU58474 receptor-like protein kinase 1 |
| *Solyc05g051240* | SNP | 62,371,312 | DOCET, JAG8810 | missense variant | Eukaryotic aspartyl protease family protein |
| *Solyc05g053650* | SNP | 64,563,634 | E42 | missense variant | 26S proteasome non-ATPase regulatory subunit 1 |
| *Solyc05g054800* | SNP | 65,473,244 | E42 | missense variant | LEM3 (Ligand-effect modulator 3) family protein |
| *Solyc05g055340* | SNP | 65,869,356 | E42 | missense variant | Plant tudor-like RNA-binding protein |
| *Solyc06g005880* | InDel | 887,895 | JAG8810 | missense variant | Non-specific serine/threonine protein kinase |
| *Solyc06g006020* | SNP | 1,039,101 | JAG8810 | missense variant | Non-specific serine/threonine protein kinase |
|  | SNP | 1,039,114 | JAG8810 | missense variant |  |
| *Solyc06g011663* | InDel | 11,054,653 | E17, E53, E76, E107, DOCET, JAG8810 | missense variant | Beta glucosidase 25 |
| *Solyc06g025425* | SNP | 11,092,629 | E36, E37, E76 | missense variant | Spc97 / Spc98 family of spindle pole body (SBP) component |
| *Solyc06g024386* | SNP | 11,253,638 | E17, E107 | missense variant | Alpha-mannosidase |
| *Solyc06g024203* | SNP | 12,304,050 | E8, E17, E36, E42, E45,  E53, E76, JAG8810 | missense variant | Peroxidase superfamily protein |
| *Solyc06g050390* | SNP | 33,086,919 | E7, E8, E17, E36, E37, E45, E53, E76, E107, DOCET, JAG8810 | missense variant | Serine/threonine-protein phosphatase 7 long form-like protein |
|  | InDel | 33,086,960 | E8, E36, E37, E45, E53, E76, DOCET, JAG8810 | missense variant |  |
| *Solyc06g066860* | InDel | 42,169,061 | E8, E53 | missense variant | 2-oxoglutarate-dependent dioxygenase-related family protein |
|  | SNP | 42,169,064 | E8, E53 | missense variant |  |
| *Solyc07g004993* | InDel | 4,480 | E42, E76 | missense variant | Phosphatidylinositol N-acetyglucosaminlytransferase subunit P-like protein |
|  | SNP | 4,482 | E42 | missense variant |  |
|  | InDel | 4,487 | E17, E42, E76, DOCET | missense variant |  |
|  | InDel | 4,511 | E8, E17, E36, E37, E42, E45, E76, DOCET, JAG8810 | missense variant |  |
|  | InDel | 4,528 | E17, E42, E76, DOCET | missense variant |  |
| *Solyc07g005215* | SNP | 206,443 | E42 | missense variant | Signal peptide peptidase-like 2 |
| *Solyc07g005250* | SNP | 220,888 | E42 | missense variant | Auxin response factor 6 |
| *Solyc07g005530* | SNP | 427,539 | E42 | missense variant | Ubiquitin carboxyl-terminal hydrolase |
| *Solyc07g008960* | SNP | 3,989,239 | E42 | missense variant | Zinc finger MYM-type-like protein |
| *Solyc07g017510* | SNP | 7,403,669 | E42 | missense variant | 1-phosphatidylinositol-3-phosphate 5-kinase |
| *Solyc07g017575* | SNP | 7,582,254 | E42 | missense variant | Flavin-containing monooxygenase |
| *Solyc07g018350* | SNP | 10,384,509 | E42 | missense variant | Mismatch repair protein |
| *Solyc07g019573* | SNP | 12,298,817 | E42 | missense variant | Guanine nucleotide-binding protein subunit beta-like protein |
|  | SNP | 12,299,069 | E42 | missense variant |  |
|  | SNP | 12,299,098 | E42 | missense variant &  splice region variant |  |
| *Solyc07g021170* | SNP | 16,613,914 | E42 | missense variant &  splice region variant | Hyp O-arabinosyltransferase-like protein |
| *Solyc07g021370* | SNP | 17,487,370 | E42 | missense variant | DNA-directed DNA polymerase |
| *Solyc07g021415* | SNP | 17,989,757 | E42 | missense variant | Serine/threonine protein phosphatase 7 long form isogeny |
| *Solyc07g021540* | SNP | 19,302,189 | E42 | missense variant | GRAM domain protein/ABA-responsive-like protein |
| *Solyc07g032300* | SNP | 37,349,864 | E42 | missense variant | Subtilisin-like protease |
| *Solyc07g037940* | SNP | 44,352,873 | E42 | missense variant | Ulp1 protease family |
| *Solyc07g039287* | SNP | 46,999,360 | E42 | missense variant | protein kinase family protein |
|  | SNP | 46,999,390 | E42 | missense variant |  |
| *Solyc07g041100* | SNP | 51,943,027 | E42 | missense variant | Structural maintenance of chromosomes (SMC) family protein |
| *Solyc07g041820* | SNP | 54,287,726 | E42 | missense variant | Ulp1 protease family |
|  | SNP | 54,287,791 | E42 | missense variant |  |
| *Solyc07g042660* | SNP | 56,308,233 | E42 | missense variant | SNF2 domain-containing protein |
|  | SNP | 56,308,257 | E42 | missense variant |  |
|  | SNP | 56,308,385 | E42 | missense variant |  |
| *Solyc07g043310* | SNP | 57,122,076 | E42 | missense variant | SNF2 domain-containing protein |
| *Solyc07g045613* | SNP | 58,909,918 | E53 | missense variant | Gamma-subunit 1 |
| *Solyc07g047950* | SNP | 59,301,795 | E42 | missense variant | GRAS family transcription factor |
| *Solyc07g051970* | SNP | 60,657,874 | E42, DOCET | missense variant | Helicase protein with RING/U-box domain-containing protein |
| *Solyc07g053010* | SNP | 61,555,808 | E42 | missense variant | NBS-LRR type disease resistance protein |
| *Solyc07g053300* | SNP | 61,899,493 | E42 | missense variant | ABC transporter family protein |
| *Solyc07g053340* | SNP | 61,924,759 | E42 | missense variant | F-box protein interaction domain protein |
| *Solyc07g053640* | InDel | 62,220,133 | E42 | disruptive inframe insertion | Arabinogalactan-protein |
| *Solyc07g053960* | SNP | 62,485,282 | E42 | missense variant | Ubiquitin conjugating enzyme |
|  | SNP | 62,485,578 | E42 | missense variant |  |
| *Solyc07g056020* | SNP | 64,052,338 | E42 | missense variant | Translation initiation factor IF-2 |
| *Solyc07g061880* | SNP | 64,903,693 | E42 | missense variant | Retrovirus-related Pol polyprotein from transposon TNT 1-94 |
| *Solyc07g062930* | SNP | 65,687,262 | E42 | missense variant | Protein methyltransferase PrmA-like |
| *Solyc07g064400* | SNP | 66,709,159 | E42 | missense variant | Serine/threonine protein phosphatase 7 long form isogeny |
|  | SNP | 66,709,426 | E42 | missense variant |  |
| *Solyc07g065300* | SNP | 67,249,290 | E42 | missense variant | Calcium-transporting ATPase |
|  | SNP | 67,249,631 | E42 | missense variant |  |
|  | SNP | 67,249,640 | E42 | missense variant |  |
|  | SNP | 67,249,681 | E42 | missense variant |  |
| *Solyc07g065870* | SNP | 67,594,035 | E42 | missense variant | Regulatory protein recX |
| *Solyc08g006252* | InDel | 933,706 | E17, E45, E107, DOCET | missense variant | BR enhanced expression 1 |
| *Solyc08g016793* | SNP | 9,434,224 | E8, E17, E36, E45, E76, JAG8810 | missense variant | F-box SKIP17-like protein |
| *Solyc08g079260* | SNP | 62,989,266 | E7, E36, E37, E45, E76, E107, JAG8810 | missense variant | Tetratricopeptide repeat-containing family protein |
| *Solyc09g055810* | SNP | 45,628,505 | E7, E37, E42, E45, E53, E76, E107, DOCET, JAG8810 | missense variant | Protein transport protein sec23 |
| *Solyc09g064590* | SNP | 62,141,923 | E7 | missense variant | Pre-mRNA-processing factor 39 |
| *Solyc09g075510* | SNP | 67,650,894 | DOCET | missense variant | Dehydration-induced 19-like protein |
| *Solyc09g092550* | SNP | 72,079,351 | E107 | missense variant | 30S ribosomal S5 |
| *Solyc10g018830* | SNP | 10,679,911 | E107 | missense variant | UDP-Glycosyltransferase superfamily protein |
| *Solyc10g044430* | SNP | 26,314,003 | E7, E17, E36, E37, E42, E45, E53, E76, DOCET, JAG8810 | missense variant | FMN-linked oxidoreductases superfamily protein |
| *Solyc10g047110* | SNP | 39,781,752 | E107 | missense variant | Peroxidase |
| *Solyc10g050100* | SNP | 47,723,097 | E17, E42, E107, DOCET, JAG8810 | missense variant | Replication factor C subunit 2 |
| *Solyc10g054950* | SNP | 56,109,088 | E17, E42 | missense variant &  splice region variant | Mediator of RNA polymerase II transcription subunit 20-like protein |
| *Solyc10g055020* | SNP | 56,185,253 | E17, E42 | missense variant | Mediator of RNA polymerase II transcription subunit 20-like protein |
| *Solyc10g077000* | SNP | 60,028,784 | E8, E42, E53 | missense variant | Transmembrane protein |
| *Solyc10g082065* | SNP | 63,080,092 | E17, E36, E37, E42, E53, JAG8810 | missense variant | Nuclear transport factor 2 family protein with RNA binding domain isoform 2 |
|  | InDel | 63,080,096 | E17, E36, E37, E42, E53, JAG8810 | missense variant |  |
| *Solyc11g005100* | SNP | 84,748 | E42 | missense variant | NAD kinase 2 |
| *Solyc11g006420* | SNP | 1,131,318 | E42 | missense variant | Cytosolic 5-nucleotidase |
| *Solyc11g006650* | SNP | 1,259,817 | E42 | missense variant | Double Clp-N motif-containing P-loop nucleoside triphosphate hydrolases superfamily protein |
|  | SNP | 1,259,910 | E42 | missense variant |  |
| *Solyc11g006950* | SNP | 1,452,986 | E42 | missense variant | Defensin-like family protein |
| *Solyc11g007010* | SNP | 1,504,997 | E42 | missense variant | Proline-, glutamic acid- and leucine-rich protein 1 |
| *Solyc11g007280* | SNP | 1,664,383 | E42 | missense variant | Pleiotropic drug resistance ABC transporter |
| *Solyc11g007320* | SNP | 1,701,960 | E42 | missense variant | Galactose oxidase/kelch repeat protein |
| *Solyc11g007370* | SNP | 1,730,322 | E42 | missense variant | Glycosyltransferase |
|  | InDel | 1,730,527 | E42 | missense variant |  |
|  | SNP | 1,730,531 | E42 | missense variant |  |
| *Solyc11g007700* | SNP | 1,939,822 | E42 | missense variant | S-adenosyl-L-methionine-dependent methyltransferase superfamily protein |
| *Solyc11g007780* | SNP | 2,022,756 | E42 | missense variant | SEC12-like protein 1 |
| *Solyc11g011500* | SNP | 4,556,122 | E7, E8, E17, E36, E37, E42, E45, E53, E76, E107, JAG8810 | missense variant | Potassium channel |
| *Solyc11g018857* | SNP | 9,719,419 | E42, JAG8810 | missense variant | Adenylate isopentenyltransferase |
|  | SNP | 9,719,516 | E42, JAG8810 | missense variant |  |
| *Solyc11g020290* | SNP | 10,719,129 | JAG8810 | missense variant | Phosphoenolpyruvate carboxylase |
|  | SNP | 10,719,281 | JAG8810 | missense variant |  |
| *Solyc11g020410* | InDel | 11,064,613 | JAG8810 | missense variant | Adenylate isopentenyltransferase |
|  | SNP | 11,064,624 | JAG8810 | missense variant |  |
| *Solyc11g020356* | SNP | 11,093,757 | JAG8810 | missense variant | Adenylate isopentenyltransferase |
| *Solyc11g020358* | SNP | 11,099,862 | JAG8810 | missense variant | Adenylate isopentenyltransferase |
| *Solyc11g020493* | SNP | 11,363,757 | JAG8810 | missense variant | Adenylate isopentenyltransferase |
|  | InDel | 11,363,821 | JAG8810 | missense variant |  |
|  | SNP | 11,364,079 | JAG8810 | missense variant |  |
| *Solyc11g020496* | SNP | 11,368,565 | E37, JAG8810 | missense variant &  splice region variant | Adenylate isopentenyltransferase |
|  | SNP | 11,368,642 | JAG8810 | missense variant |  |
| *Solyc11g020498* | SNP | 11,368,884 | JAG8810 | missense variant | Adenylate isopentenyltransferase |
| *Solyc11g021310* | SNP | 13,420,419 | E7, E8, E36, E107 | missense variant | Ycf1 |
| *Solyc11g030910* | InDel | 23,584,775 | E7 | missense variant | Leucine-rich repeat protein kinase family protein |
| *Solyc11g044840* | SNP | 32,479,526 | E42, JAG8810 | missense variant | Aspartate aminotransferase |
| *Solyc11g073270* | SNP | 56,573,865 | E36, E37 | missense variant | Pentatricopeptide repeat-containing protein |
| *Solyc12g005770* | InDel | 415,671 | E107 | conservative inframe deletion | Zinc finger transcription factor 71 |
| *Solyc12g008420* | SNP | 1,847,010 | E42 | missense variant | RNA-dependent RNA polymerase |
| *Solyc12g011400* | SNP | 4,221,130 | E42 | missense variant | Pentatricopeptide repeat-containing protein |
| *Solyc12g017230* | SNP | 6,423,168 | E42 | missense variant | Modifier of snc1 |
| *Solyc12g021210* | SNP | 14,818,293 | E42 | missense variant | E3 ubiquitin protein ligase DRIP2 |
| *Solyc12g082750* | SNP | 16,912,003 | E42 | missense variant | CRS1/YhbY (CRM) domain-containing protein |
|  | SNP | 16,912,032 | E42 | missense variant |  |
| *Solyc12g082770* | SNP | 17,451,774 | E42 | missense variant | Pyruvate orthophosphate dikinase |
| *Solyc12g076355* | SNP | 22,638,921 | E7, E17, E76, E107 | missense variant | 3-oxo-5-alpha-steroid 4-dehydrogenase family protein |
|  | SNP | 22,639,762 | E7, E8, E17, E36, E37, E45, E53, E76, E107, DOCET, JAG8810 | missense variant |  |
|  | InDel | 22,638,927 | E7, E8, E17, E37, E53, DOCET, JAG8810 | missense variant |  |
| *Solyc12g062560* | SNP | 33,928,024 | E42 | missense variant | Ribulose bisphosphate carboxylase large chain |
| *Solyc12g041980* | SNP | 56,789,571 | E42 | missense variant | Protein Breast cancer susceptibility-like 1 |
| *Solyc12g042025* | SNP | 57,579,211 | E42 | missense variant | 2-oxoglutarate (2OG and FeII)-dependent oxygenase superfamily protein |
|  | SNP | 57,579,218 | E42 | missense variant |  |
|  | SNP | 57,579,227 | E42 | missense variant |  |
| *Solyc12g043090* | SNP | 59,689,826 | E42 | missense variant | Transcription factor |
|  | SNP | 59,689,905 | E42 | missense variant |  |
