# Supplementary material for: High-Throughput Genotyping of Resilient Tomato Landraces to Detect Candidate Genes Involved in the Response to High Temperatures
Source: Genes (Basel). 2020 Jun 7;11(6):626. doi: 10.3390/genes11060626 (PMC7349060; doi:10.3390/genes11060626)
Supplement: Supplementary file 1 [file genes-11-00626-s001.zip › Supplementary material/Supplementary Table S8.docx]

**Supplementary Table S8** Percentage of identity of 63 private InDels for E42 with three wild tomato species. Markers coded S are SSR markers and coded N are InDels. For each mutation, the percentage of identity and the position on scaffolds/contigs are reported for the related wild species genomes.

| Code | Chr | Reference allele | E42 allele | *S. lycopersicum* | | | *Wild species* | | | |
| --- | --- | --- | --- | --- | --- | --- | --- | --- | --- | --- |
|  |  |  |  | Position | Identities | | Species | Identities | | Homologous |
| N1 | 1 | AC | ACGGTACTC | 2,372,368 | 101/108 | (94%) | *S. chilense* | 109/110 | (99%) | scaffold 965 |
| S1 | 1 | A(AT)_9_C | A(AT)_8_C | 2,974,473 | 99/101 | (98%) | *S. pimpinellifolium* | 99/99 | (100%) | contig 3056440 |
| S2 | 1 | G(CA)_6_T | GCG(CA)_5_T | 22,951,379 | 100/101 | (99%) | *S. pimpinellifolium* | 101/101 | (100%) | contig 6542704 |
| N2 | 1 | CT | CTTTCAAGGATTTT | 26,813,774 | 101/113 | (89%) | *S. pimpinellifolium* | 89/89 | (100%) | contig 6488329 |
| N3 | 1 | TG | TGTGTGGTAATAG | 30,621,680 | 101/112 | (90%) | *S. pimpinellifolium* | 112/112 | (100%) | contig 6652606 |
| N4 | 1 | TT | TTAAGT | 36,554,244 | 101/105 | (96%) | *S. chilense* | 106/109 | (97%) | scaffold 3237 |
| S3 | 1 | T(ATA)_6_A | T(ATA)_5_A | 44,922,272 | 98/101 | (97%) | *S. pimpinellifolium* | 98/98; 98/98; 98/98. | (100%); (100%); (100%). | contig 6610437; contig 6533408; contig 6429843. |
| S4 | 1 | G(TGATAA)_3_T | GTGATAAT | 58,297,766 | 89/101 | (88%) | *S. pimpinellifolium* | 84/86; 83/86. | (97%); (96%). | contig 6562334; contig 6493411. |
| N5 | 1 | CA | CAAATCGAGCCCTA | 60,913,274 | 101/113 | (89%) | *S. pimpinellifolium* | 77/77 | (100%) | contig 6494590 |
| S5 | 1 | T(AG)_8_G | T(AG)_9_G | 67,567,837 | 101/103 | (98%) | *S. pimpinellifolium* | 102/103 | (99%) | contig 6739562 |
| S6 | 1 | TTAA | T(TA)_2_A | 67,778,204 | 101/103 | (98%) | *S. pimpinellifolium* | 102/103 | (99%) | contig 6545439 |
| S7 | 1 | C(GT)_3_C | C(GT)_2_C | 73,088,727 | 99/101 | (98%) | *S. pimpinellifolium* | 98/99 | (98%) | contig 6448937 |
| S8 | 1 | TT | T(TGG)_2_T | 75,154,593 | 101/107 | (94%) | *S. pimpinellifolium* | 102/108 | (94%) | contig 6590182 |
| N6 | 1 | AATAGAATGGAAGCA | AA | 79,508,022 | 75/87 | (87%) | *S. pimpinellifolium* | 87/87 | (100%) | contig 6604148 |
| S9 | 1 | T(ATA)_2_A | TATAA | 79,746,712 | 98/101 | (97%) | *S. pimpinellifolium* | 94/98 | (95%) | contig 6623428 |
| S10 | 1 | ATT(CTTTCTT)_5_ | ATT(CTTTCTT)_6_ | 80,392,618 | 103/106 | (97%) | *S. pimpinellifolium* | 108/108 | (100%) | contig 6686636 |
| N7 | 1 | CAAGGAA | CA | 80,981,869 | 90/95 | (95%) | *S. pimpinellifolium* | 73/75 | (97%) | contig 2506929 |
| N8 | 1 | AGCATTC | AGCATTGCATTC | 81,916,254 | 101/106 | (95%) | *S. pimpinellifolium* | 106/106 | (100%) | contig 6453285 |
| N9 | 1 | CGC | TGAAATA | 83,380,952 | 99/105 | (94%) | *S. pimpinellifolium* | 105/105 | (100%) | contig 3764285 |
| N10 | 1 | TA | TATAAAGAGGTCTTGTAGACA | 83,585,992 | 101/120 | (84%) | *S. pimpinellifolium* | 111/120 | (93%) | contig 6576863 |
| S11 | 2 | TT(ATAGAAT)_2_ | TTATAAGAAT | 50,399,678 | 93/101 | (92%) | *S. pimpinellifolium* | 93/93 | (100%) | contig 6605169 |
| S12 | 2 | T(TC)_6_T | T(TC)_5_T | 50,655,311 | 99/101 | (98%) | *S. pimpinellifolium* | 99/99; 93/93 | (100%); (100%). | contig 3329377; contig 6605169. |
| N11 | 4 | CCG | ACGAAAATGTTGATG | 5,212,435 | 99/112 | (88%) | *S. chilense* | 114/116 | (98%) | scaffold 8444 |
| N12 | 4 | CCCTTCCTTA | CCCTTA | 5,479,048 | 93/96 | (96%) | *S. galapagense* | 114/114 | (100%) | scaffold 7185_11.6_contig3 |
| N13 | 4 | AGCG(TGTGCG)_3_ | (CG)_2_(TG)_2_CGTGAGCG(TG)_2_ | 8,543,456 | 99/101 | (98%) | *S. pimpinellifolium* | 95/96; 95/96. | (98%); (98%). | contig 6674052; contig 6527707. |
| N14 | 4 | CTCCTATGAACATAG | CA | 19,392,294 | 75/87 | (86%) | *S. pimpinellifolium* | 86/87 | (99%) | contig 6600672 |
| N15 | 4 | AAAGATACAGTTTATTTTA | AA | 20,394,303 | 70/84 | (83%) | *S. pimpinellifolium* | 80/81 | (99%) | contig 6727529 |
| S13 | 4 | AA | A(CAAAAA)_2_AAGA | 25,313,477 | 103/116 | (89%) | *S. chilense*; *S. galapagense* | 114/117; 116/117. | (97%); (99%). | scaffold 1300; scaffold 18424_14.5_contig12. |
| N16 | 4 | GC | GCTGAGCC | 35,743,428 | 101/107 | (94%) | *S. galapagense* | 108/108 | (100%) | scaffold 123077_14.6 |
| N17 | 4 | AA | AACCCTGA | 46,835,168 | 101/107 | (94%) | *S. galapagense* | 107/108 | (99%) | scaffold 114129__8.6_contig1 |
| N18 | 4 | CAACAAAGAAAATGAATAAATCTCTGAA | CA | 59,202,193 | 64/68 | (94%) | *S. pimpinellifolium* | 74/74 | (100%) | contig 6576309 |
| N19 | 4 | ATGACTTGATTT | AT | 61,888,529 | 82/91 | (90%) | *S. galapagense* | 93/95 | (98%) | scaffold 103684__4.1_contig1 |
| S14 | 6 | TATATA | GATGTC | 11,130,942 | 98/101 | (97%) | *S. galapagense* | 96/100 | (96%) | C8774894_62.0 |
| N20 | 7 | TAAGTTTACTTCAGCA | TA | 575,335 | 75/87 | (86%) | *S. pimpinellifolium* | 87/87 | (100%) | contig 5133554 |
| S15 | 7 | C(AT)_6_A | C(AT)_5_A | 1,367,625 | 99/101 | (98%) | *S. pimpinellifolium* | 98/99 | (98%) | contig 313943 |
| N21 | 7 | AT | AGTT(GT)_2_ | 3,090,132 | 101/107 | (94%) | *S. pimpinellifolium* | 107/107 | (100%) | contig 711842 |
| S16 | 7 | AA(ACA)_3_ACT | AA(ACA)_4_ACT | 9,169,255 | 101/104 | (97%) | *S. pimpinellifolium* | 103/104 | (99%) | contig 6710814 |
| S17 | 7 | A(AC)_3_A | A(AC)_4_A | 9,607,725 | 101/103 | (98%) | *S. pimpinellifolium* | 103/103 | (100%) | contig 6466412 |
| S18 | 7 | A(TG)_6_T | A(TG)_7_T | 12,883,701 | 101/103 | (98%) | *S. pimpinellifolium* | 103/103 | (100%) | contig 6430357 |
| N22 | 7 | TA | TA(TG)_4_CAA | 17,910,651 | 106/112 | (95%) | *S. galapagense* | 113/113 | (100%) | scaffold 1541_15.3_contig2 |
| S19 | 7 | T(AC)_6_A | T(AC)_5_A | 21,684,017 | 99/101 | (98%) | *S. pimpinellifolium* | 73/78; 85/94. | (93%); (90%). | contig 6581372; contig 6475502. |
| S20 | 7 | G(TA)_4_T | G(TA)_6_T | 23,493,276 | 101/105 | (96%) | *S. pimpinellifolium* | 103/105 | (98%) | contig 6734913 |
| N23 | 7 | GA | GACATGCTTAGTA | 24,101,114 | 98/112 | (88%) | *S. galapagense* | 113/113 | (100%) | C9741765_11.0 |
| N24 | 7 | TT | TTTGC(AT)_2_ | 25,200,096 | 101/108 | (94%) | *S. galapagense* | 107/109 | (98%) | scaffold 18002_14.3_contig4 |
| S21 | 7 | GTATC | (AT)_2_G | 50,363,979 | 99/101 | (98%) | *S. pimpinellifolium* | 97/101 | (96%) | contig 6711062 |
| S22 | 7 | A(AT)_3_C | AATC | 51,299,430 | 97/101 | (96%) | *S. pimpinellifolium* | 97/97 | (100%) | contig 2014799 |
| S23 | 7 | AGCG | A(GC)_2_G | 57,711,705 | 101/103 | (98%) | *S. pimpinellifolium* | 103/104 | (99%) | contig 6585467 |
| S24 | 7 | G(CT)_13_A | G(CT)_14_ | 59,089,857 | 100/103 | (97%) | *S. pimpinellifolium* | 82/82 | (100%) | contig 6605025 |
| N25 | 7 | TCCA(AC)_2_CATCAC | TCCA(AC)_2_CATCACCAACACCATCAC | 62,220,133 | 101/113 | (89%) | *S. pimpinellifolium* | 113/113 | (100%) | contig 442915 |
| N26 | 7 | TGGCA | AGGCAA(GT)_2_AGTAA | 63,166,300 | 101/111 | (91%) | *S. pimpinellifolium* | 78/78 | (100%) | contig 5929686 |
| N27 | 7 | TA | TAAA(TA)_2_CGATGAAA | 63,747,960 | 101/115 | (88%) | *S. pimpinellifolium* | 115/115 | (100%) | contig 6713826 |
| N28 | 7 | TG | (TTA)_2_ATTAATCCAG | 65,953,381 | 101/115 | (88%) | *S. pimpinellifolium* | 113/115 | (98%) | contig 2156949 |
| S25 | 7 | AT(ATT)_6_ATG | AT(ATT)_5_ATG | 66,083,815 | 98/101 | (97%) | *S. pimpinellifolium* | 98/98 | (100%) | contig 1914225 |
| N29 | 7 | CATATTTAGT | CT | 67,320,351 | 84/93 | (92%) | *S. pimpinellifolium* | 90/93 | (97%) | contig 2357274 |
| N30 | 7 | CATGATACCTT | CATGATACCTATGATACCTT | 68,142,689 | 101/110 | (92%) | *S. chilense*; *S. galapagense* | 111/112; 111/111. | (99%); (100%). | scaffold 4; scaffold 45374__9.1. |
| N31 | 8 | TA | TTAAATAGA | 60,993,787 | 101/108 | (94%) | *S. pimpinellifolium* | 107/108 | (99%) | contig 6741274 |
| S26 | 10 | AT(ATT)_2_ | AT(ATT)_3_ | 63,052,932 | 101/104 | (97%) | *S. pimpinellifolium* | 104/104 | (100%) | contig 979111 |
| N32 | 11 | CA | CATGTCATGATTA | 1,733,683 | 100/111 | (90%) | *S. pimpinellifolium* | 94/95 | (99%) | contig 6620295 |
| S27 | 11 | T(AGTTAGTTA)_2_G | TAGGTAGTTAG | 53,542,500 | 92/101 | (91%) | *S. pimpinellifolium* | 92/92 | (100%) | contig 6614407 |
| N33 | 12 | TA | TA(CT)_2_AATACAAAATCA | 25,738,369 | 101/117 | (86%) | *S. galapagense* | 115/118 | (97%) | scaffold 66473_16.4_contig1 |
| N34 | 12 | CT | CTAGAACTTCAAT | 35,054,202 | 95/112 | (85%) | *S. galapagense* | 113/113 | (100%) | scaffold 47841_16.4_contig2 |
| N35 | 12 | GC | GAAAATGGGAG | 38,273,874 | 100/110 | (91%) | *S. galapagense* | 111/111 | (100%) | scaffold 21937_16.7_contig2 |
| N36 | 12 | AT | ATTAAAGTTTGCT | 59,496,533 | 100/111 | (90%) | *S. pimpinellifolium* | 108/112 | (96%) | contig 6689634 |
